# The Increasing Trend in Caesarean Section Rates: Global, Regional and National Estimates: 1990-2014

**DOI:** 10.1371/journal.pone.0148343

**Published:** 2016-02-05

**Authors:** Ana Pilar Betrán, Jianfeng Ye, Anne-Beth Moller, Jun Zhang, A. Metin Gülmezoglu, Maria Regina Torloni

**Affiliations:** 1 UNDP/UNFPA/UNICEF/WHO/World Bank Special Programme of Research, Development and Research Training in Human Reproduction (HRP), Department of Reproductive Health and Research, World Health Organization, Geneva, Switzerland; 2 Department of Clinical Epidemiology, Obstetrics and Gynecology Hospital, Fudan University, Shanghai, China; 3 Ministry of Education—Shanghai Key Laboratory of Children’s Environmental Health, Xinhua Hospital, Shanghai Jiao Tong University School of Medicine, Shanghai, China; 4 Evidence Based Healthcare Post-Graduate Program, Department of Medicine, São Paulo Federal University, São Paulo, Brazil; Leibniz Institute for Prevention Research and Epidemiology (BIPS), GERMANY

## Abstract

**Background:**

Caesarean section (CS) rates continue to evoke worldwide concern because of their steady increase, lack of consensus on the appropriate CS rate and the associated additional short- and long-term risks and costs. We present the latest CS rates and trends over the last 24 years.

**Methods:**

We collected nationally-representative data on CS rates between 1990 to 2014 and calculated regional and subregional weighted averages. We conducted a longitudinal analysis calculating differences in CS rates as absolute change and as the average annual rate of increase (AARI).

**Results:**

According to the latest data from 150 countries, currently 18.6% of all births occur by CS, ranging from 6% to 27.2% in the least and most developed regions, respectively. Latin America and the Caribbean region has the highest CS rates (40.5%), followed by Northern America (32.3%), Oceania (31.1%), Europe (25%), Asia (19.2%) and Africa (7.3%). Based on the data from 121 countries, the trend analysis showed that between 1990 and 2014, the global average CS rate increased 12.4% (from 6.7% to 19.1%) with an average annual rate of increase of 4.4%. The largest absolute increases occurred in Latin America and the Caribbean (19.4%, from 22.8% to 42.2%), followed by Asia (15.1%, from 4.4% to 19.5%), Oceania (14.1%, from 18.5% to 32.6%), Europe (13.8%, from 11.2% to 25%), Northern America (10%, from 22.3% to 32.3%) and Africa (4.5%, from 2.9% to 7.4%). Asia and Northern America were the regions with the highest and lowest average annual rate of increase (6.4% and 1.6%, respectively).

**Conclusion:**

The use of CS worldwide has increased to unprecedented levels although the gap between higher- and lower-resource settings remains. The information presented is essential to inform policy and global and regional strategies aimed at optimizing the use of CS.

## Introduction

A caesarean section (CS) is a life-saving surgical procedure when certain complications arise during pregnancy and labour. However, it is a major surgery and is associated with immediate maternal and perinatal risks and may have implications for future pregnancies as well as long-term effects that are still being investigated [1–4]. The use of CS has increased dramatically worldwide in the last decades particularly in middle- and high-income countries, despite the lack of evidence supporting substantial maternal and perinatal benefits with CS rates higher than a certain threshold, and some studies showing a link between increasing CS rates and poorer outcomes [5, 6]. The reasons for this increase are multifactorial and not well-understood. Changes in maternal characteristics and professional practice styles, increasing malpractice pressure, as well as economic, organizational, social and cultural factors have all been implicated in this trend [7–10]. Additional concerns and controversies surrounding CS include inequities in the use of the procedure, not only between countries but also within countries and the costs that unnecessary caesarean sections impose on financially stretched health systems [11, 12].

Country-level CS rates worldwide were compiled and global and regional estimates were generated and published in 2007 [13]. The objective of our analysis is to update previous published estimates, present the latest data on national CS rates worldwide and to analyze trends over the last decades.

## Materials and Methods

### Source of Caesarean Section Rates at National Level

The rate of CS is expressed as a percentage calculated by dividing the number of caesarean deliveries over the total number of live births. We obtained the rates of CS from three sources: i) nationally representative surveys, ii) routine vital statistics, and iii) reports from health authorities. See S1 File for the first and latest available CS rate data points per country, the year, total number of data points used for this analysis and sources of the data.

For developing countries, we obtained data mainly using the Demographic and Health Surveys (DHS) Program [14] and the Multiple Indicator Cluster Surveys (MICS) [15]. Since 1984, the DHS MACRO program has earned a worldwide reputation for collecting and disseminating accurate, nationally representative data on maternal and infant health and nutrition in more than 300 surveys in over 90 countries. The MICS programme started in 1995 mainly in countries not covered under the DHS program and has become an important source of statistically sound and comparable data since then and over 300 surveys in 100 countries have been conducted to date. In both programmes, surveys are conducted by trained personnel using standardised questionnaires and strict methods for data collection and processing. These surveys are considered the best available way of obtaining several types of health indicators in developing countries and the reliability of reported national rates of CS has been recognized [16]. As these surveys are typically conducted about every 5 years, comparisons over time are possible and desirable. The figures for CS rates obtained through the DHS refer to births that occurred between 3 to 5 years previous to the date of the survey; in the MICS, they refer to births occurring in the 2 previous years.

For developed countries, CS rates were obtained from vital statistics. For European countries, data were obtained from the European Health for All Database [17], maintained by the WHO European Regional Office. For the other developed countries (i.e. Australia, Canada, Japan, New Zealand and United States), data were obtained from routine statistical surveillance system or government health offices reports.

For countries without any of the above-mentioned sources, we searched for information on CS rates in publications available through electronic databases, web search engines and government web sites. Data collection was closed on 15 April 2015 and data published after this date were not included in this analysis.

### Coverage of Global and Regional Estimates

The latest available data from each country were used to calculate current global and regional rates of CS. However, if the most recent available data predated 2005, then the country was excluded from this study because we considered the data as being too old. Countries were grouped according to the United Nations’ geographical grouping [18] and regional and subregional averages for the proportion of CS were calculated as weighted means based on the country’s share of live births in 2010 in the region or subregion, respectively [19]. “Coverage” was used as a measure to express how representative an estimate is with regard to the region/subregion. Regional and subregional coverage were calculated as the proportion of total regional and subregional live births for which nationally representative data on CS were available. Estimates for subregions with a coverage less than 60% were not calculated.

### Trends Over Time Analyses

We collected data and describe trends from 1990 to 2014. Only countries with a minimum of two data points over a time span of 10 years were included in the trends analysis; thus the number of countries in the trend analysis is different from the number of countries included in the cross-sectional overview. We describe the absolute increase in CS rate per country, subregion and region. This was obtained by subtracting the earliest CS rate from the latest CS rate. We also present the rate in change in CS rates from 1990 to 2014 by region and subregion by calculating the average annual rate of increase (AARI), where AARI = [(a_n_ / a_m_) ^[1 / (n-m)]^]-1; a_m_ is the first observation of CS rate, a_n_ is the latest observation of CS rate, m is the first observed year and n is the latest observed year. The AARI is a geometric progression ratio that provides a constant rate of change during the study period. It can be interpreted as the average percent by which CS rates increased or decreased each year. The AARI of the CS rate allows comparisons with the assumption that the CS rate has changed at a constant speed over the given time period. For the trend analysis, if the data in 2014 were not available, the latest data (available from 2005 or later) were used. If the data in 1990 were not available, the closest available data from 1985–1995 were used. In case of equal closeness in both time directions, the earlier data were used. All analyses were performed using SAS statistical package version 9.2 (SAS Institute Inc., Cary, NC, USA).

As the majority of countries did not have CS rates available on yearly basis, for the purpose of graphing the trends by region, a linear interpolation between available CS rates for each country from 1990–2014 was calculated. Based on the data available, the following conservative assumptions were made: (1) If the data in 1990 were not available, all CS rates before the first available CS rate were equal to the first CS rate; (2) If the data in 2014 were not available, then all CS rates after the last available CS rate are equal to the latest CS rate; (3) Between available CS rates in each country, CS rates were assumed to have changed linearly.

## Results

### Current Rates of Caesarean Section Worldwide

Data were available for 150 countries to estimate current global and regional CS rates. This represents 96.1% of all live births worldwide in 2010 (Table 1). Coverage was high throughout the regions and subregions. At regional level, except for Oceania with 62.3% of the live births of the region represented, coverage ranged from 92.8% in Africa to 100% in Northern America. At subregional level, excluding Southern Africa where data were available for only 11.7% of the live births, coverage ranged from 81.8% in the Caribbean to 100% in eight subregions (Table 1).

**Table 1:** Caesarean section rates in 150 countries categorised according to United Nations geographical grouping in 2014^a^.

Region/subregion^b^	Births by cesarean section (%)	Range (minimum to maximum) (%)	Coverage of estimates (%)
**Africa**	7.3	1.4–51.8	92.8
Eastern Africa	3.9	1.5–9.6	96.3
Middle Africa	5.8	3.8–10.0	83.2
Northern Africa	27.8	6.6–51.8	97.4
Southern Africa^c^	-	-	-
Western Africa	3.0	1.4–11.4	100
**Asia**	19.2	1.7–47.5	97.8
Eastern Asia	34.8	12.5–36.6	100
South-central Asia	11.4	3.6–47.9	100
South-eastern Asia	14.8	1.7–32.0	91.4
Western Asia	26.8	4.8–47.5	87.4
**Europe**	25.0	13.9–38.1	98.6
Eastern Europe	23.7	15.8–36.3	100
Northern Europe	22.4	14.7–26.6	100
Southern Europe	30.7	13.9–38.1	92.7
Western Europe	24.5	15.6–32.2	100
**Latin America and the Caribbean**	40.5	5.5–55.6	93.7
Caribbean	27.5	5.5–53.4	81.8
Central America	38.2	16.3–45.2	100
Southern America	42.9	13.3–55.6	91.7
**Northern America**	32.3	27.1–32.8	100
**Oceania**	31.1	6.2–33.4	62.3
Australia/New Zealand	32.3	32.4–33.4	100
**World total**^**b**^	**18**.**6**	**1**.**4–56**.**4**	**96**.**1**
Least developed regions	6.0	1.4–41.1	91.8
Less developed regions	20.9	1.7–56.4	96.9
More developed regions	27.2	13.9–38.1	99.2

^a^ If the data in 2014 was not available, the latest data available from 2005 was used instead.

^b^ Countries categorized according to the UN geographical grouping. Number of live births in each country in 2010 was used as a weight to calculate the regional coverage.

^c^ Estimates for subregions with a coverage less than 60% are not calculated. Coverage for Southern Africa is 11.7%.

According to the most recent estimates, the average global rate of CS is 18.6%, ranging from 6.0% to 27.2% in the least and more developed regions, respectively (Table 1). The lowest rates of CS are found in Africa (7.3%) and more specifically in Western Africa (3%). The highest rates of CS are found in Latin American and the Caribbean (40.5%) and South America is the subregion with the highest average CS rates in the world (42.9%).

**Fig 1:**
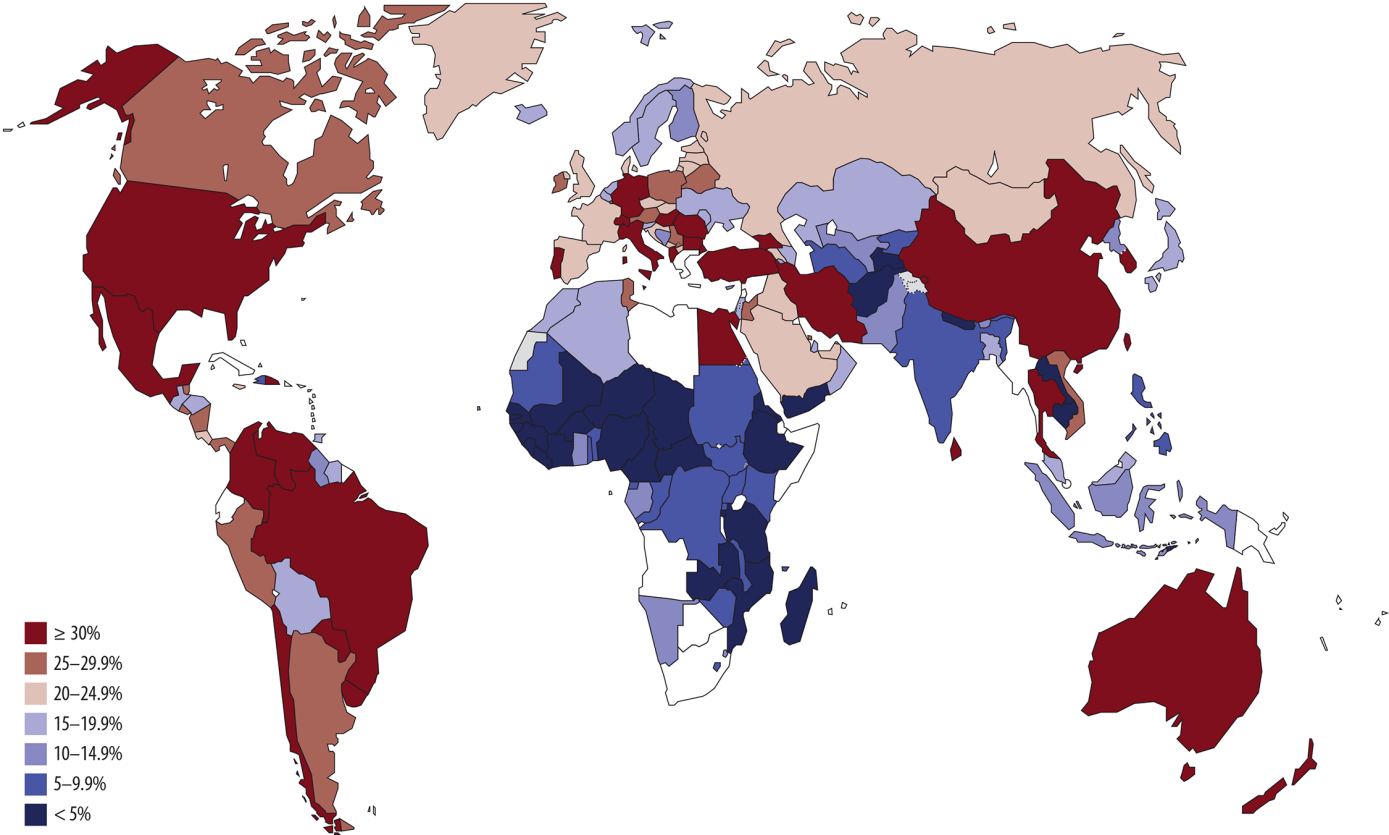
Latest available data on caesarean section rates by country (not earlier than 2005).

Countries with the highest CS rates in each region are Brazil (55.6%) and Dominican Republic (56.4%) in Latin America and the Caribbean, Egypt (51.8%) in Africa, Iran and Turkey in Asia (47.9% and 47.5%, respectively), Italy (38.1%) in Europe, United States (32.8%) in Northern America, and New Zealand (33.4%) in Oceania (S1 File). Fig 1 shows country variation of CS rates according to latest nationally-representative reported data.

### Trend in Rates of Caesarean Section

Table 2 shows trends in CS rates from 1990 to 2014 by region and subregion. This trend analysis included 121 countries representing 90% of the total number of live births worldwide. During this period of time, CS rates decreased only in two countries: Guinea, from 3.3% to 2.4%; and Nigeria, from 2.9% to 2%. Zimbabwe maintained the rate at 6% and all other countries increased their use of CS (S1 File). The change in the rate of CS in Table 2 is presented as absolute increase as well as the AARI.

**Table 2:** Change in caesarean section rates in 121 countries categorised according to the United Nations geographical grouping from 1990 to 2014^a^.

Region/subregion^b^ (coverage, %)	Change in rate (earliest and latest rates, %)	Absolute increase (%)	AARI^c^ (% per year)
**Africa (81.8)**	**2**.**9–7**.**4**	**4**.**5**	**4**.**0**
Eastern Africa (96.3)	2.3–3.9	1.6	2.2
Middle Africa^d^	-	-	-
Northern Africa (97.3)	4.5–27.8	23.3	7.9
Southern Africa^d^	-	-	-
Western Africa	2.6–3.1	0.5	0.7
**Asia (93.1)**	**4**.**4–19**.**5**	**15**.**1**	**6**.**4**
Eastern Asia (97.8)	4.9–35.2	30.3	8.5
South-central Asia (96.4)	4–11.4	7.4	4.4
South-eastern Asia (84.0)	4.1–15	10.9	5.5
Western Asia (68.9)	6.3–28.1	21.8	6.4
**Europe (98.1)**	**11.2–25**.**0**	**13**.**8**	**3**.**4**
Eastern Europe (100)	7.8–23.7	15.9	4.7
Northern Europe (100)	11.1–22.4	11.3	3
Southern Europe (90.3)	16.3–31.1	14.8	2.7
Western Europe (100)	14.8–24.5	9.7	2.1
**Latin America and the Caribbean (84**.**3)**	**22**.**8–42**.**2**	**19**.**4**	**2**.**6**
Caribbean (67.5)	9.9–28.5	18.6	4.5
Central America (97.9)	14.8–38.4	23.6	4.1
Southern America (79.4)	28.4–45.8	17.4	2
**Northern America (100)**	**22**.**3–32**.**3**	**10**	**1**.**6**
**Oceania (56**.**6)**	**18**.**5–32**.**6**	**14**.**1**	**2**.**4**
Australia/New Zealand (100)	18.5–32.6	14.1	2.4
**World total**^**b**^ **(90)**	**6**.**7–19**.**1**	**12**.**4**	**4**.**4**
Least developed regions (74.5)	**1**.**9–6**.**1**	4.2	5
Less developed regions (93)	**6**.**3–20**.**9**	14.6	5.1
More developed regions (98.9)	**14**.**5–27**.**2**	12.7	2.6

Change is presented as absolute increase (in percent points; latest CS rate minus earliest CS rate) and relative increase as average annual rate of increase (AARI).

^a^ If the data in 2014 is not available, the latest data (available from 2005 or later) is used instead of 2014. If the data in 1990 is not available, the earliest available data from 1985–1995 is used.

^b^ Countries categorized according to the UN geographical grouping. Number of live birth in 2000 was used as a weight to calculate the regional coverage.

^c^ AARI: average annual rate of increase = (a_m_/a_n_)^[1/(n-m)]-1. a_m_: the first observation of caesarean section rate, a_n_: the latest observation of caesarean section rate, m: the first observed year, n: the latest observed year.

^d^ Estimates for subregions with a coverage less than 60% are not calculated. Coverage for Middle Africa is 29% and for Southern Africa 4.6%.

Worldwide, CS rates increased from 6.7% in 1990 to 19.1% in 2014, which represents a 12.4% absolute increase and an AARI of 4.4%. Less developed countries showed the largest absolute increase, 14.6 points (from 6.3% to 20.9%; AARI = 5.1%). More developed countries followed with 12.7 points of absolute increase in the CS rate (from 14.5% to 27.2%; AARI = 2.6%). The rate of CS in least developed countries only rose by 4.2 points (from 1.9% to 6.1%; AARI = 5%) (Table 2).

**Fig 2:**
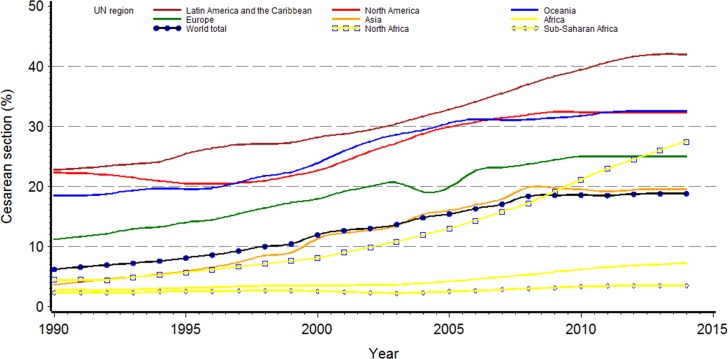
Global and regional trends in caesarean section, 1990–2014. Sub-Saharan Africa includes Eastern, Middle, Southern and Western Africa subregions. For the purpose of this graph, a linear interpolation between available data from 1990 and 2014 was calculated. When data for 2014 were not available, the CS rate for the latest year available was used also for all subsequent years up to 2014.

Table 2 and Fig 2 show global and regional trends by UN geographical grouping from 1990 to 2014. Latin America and the Caribbean which started with the highest rate in 1990 (22.8%) is also the region with the largest rate in 2014 and the largest absolute increase in CS rate (19.4 points). The region with the second largest absolute increase was Asia going from a CS rate of 4.4% in 1990 to 19.5% in the latest estimates. North America and Oceania had similar increases in their CS rates over the last 24 years (Fig 2). Among the more developed regions, Europe had the lowest CS rate throughout the time period studied. In Africa, large differences are seen between Northern Africa and the other subregions (Table 2). Given the marked difference in CS rates between the Northern Africa subregion and other subregions in Africa, in Fig 2, Eastern, Middle, Southern and Western Africa are merged and called sub-Saharan Africa and presented independently from Northern Africa. The data in Fig 2 shows how Northern Africa rose from 4.5% to 27.8%, whereas in Sub-Saharan Africa, the change in rates of CS over the past 24 years was minimal (from 2.3% to 3.5%). For the purpose of graphing the trends by region and given that data were not available on a yearly basis, a linear interpolation between available data from 1990 and 2014 was calculated. When data for 2014 were not available, the CS rate for the latest year available was used also for all subsequent years up to 2014. This conservative assumption yielded an apparent “flattening” in the increase of CS rates in the graph. This should not, however, be understood as a stabilization of CS rates.

At regional level, the largest AARI (average percentage by which CS rates increased every year) was found in Asia (6.4%), the second being Africa (4%), and the third is Europe (3.4%). At subregional level, in general, the largest yearly increases go along with the largest absolute increases. Northern Africa stands out with 23.3 points of absolute increase and 7.9% AARI; Eastern Asia surpasses the other Asian sub-regions with 30.3 points of absolute increase and 8.5% AARI; and in Europe, Eastern Europe presents both the largest absolute increase (15.9 points) and the highest AAR (4.7%).

At country level, Egypt, Tunisia and Morocco witnessed the largest rise in the African region (country-level data is shown in S1 File). CS rates in Egypt rose from 4.6% to 51.8% (47.2 points) over the 24 year period. Along with Morocco, these two countries had the largest worldwide AARI in CS rates (11.6%). In Asia, Turkey, Georgia and China had absolute increases of 39.5, 32.9, and 31.8 points, respectively and all three had an AARI of about 10%. In Europe, Romania stands out as the country with the largest absolute increase (29.1 points, from 7.2% to 36.3%). In Latin America, the three countries with the highest absolute increases in CS rates were Dominican Republic, Mexico and Colombia (36.4, 32.8, and 27.4 points, respectively).

On the other side of the spectrum, Zambia (from 2.6% to 3%), Niger (from 0.9% to 1.4%) and Burkina Faso (from 1.3% to 1.9%) were the countries with the smallest absolute increase in CS rates (only half point). In Asia, the countries with the smallest absolute increases were Cambodia, Tajikistan and Yemen with 2.2, 2.7 and 3.4 points, respectively. In Europe, Finland was the country with the smallest rise (less than 2% absolute increase) followed by Iceland and Norway with a rise of about 4.5 points. In Latin America and the Caribbean, Costa Rica and Haiti had absolute increases of CS rates below 4%. Across the world, Finland and Costa Rica were the countries with the smallest AARI in CS (0.4%) followed by Zambia (1%), Norway (1.6%) and Iceland (1.7%).

## Discussion

Over the past decades, the unprecedented and steady rise in the rates of CS have led to increased research, debate and concern among healthcare professionals, governments, policy-makers, scientists and clinicians [10, 20–23]. Despite the worldwide relevance and interest on this topic, this is the first study that presents global and regional trends in the use of CS. The latest available data show that almost 1 in 5 women in the world now give birth by CS. At present, 40.5% of all births are by CS in Latin America and the Caribbean and Southern America is the subregion with the highest rates of CS in the world with 42.9%. Africa shows the lowest average rate of CS with 7.3%, which is a weighted average between 3.5% in sub-Saharan Africa and 27.8% in Northern Africa.

The trend analyses show that except for two countries (Guinea and Nigeria) in which CS rates decreased and one (Zimbabwe) that maintained the same rate, all other countries have increased the use of CS at different levels (S1 File). The absolute and yearly increases are more remarkable in less developed countries. At the regional level, Latin America and the Caribbean had the largest absolute increase in CS rates (19.4%) which were above any other region during the whole study period (Fig 2). On the other extreme, Africa had the smallest absolute rise (4.5%) and remained as the region with the lowest CS rates throughout the study period. In particular, sub-Saharan Africa maintained virtually the same rate while the countries in Northern Africa presented a steeper increase particularly since 2000. Asia witnessed the second largest absolute increase and the largest AARI, although it is still currently the region with the second lowest CS rate in the world (19.2%), after Africa (Fig 2).

Some countries have experienced remarkable increases. Egypt, Turkey, Dominican Republic, Georgia and China have all had over 30 percent points increase in their CS rates over the last 24 years. For example, in Egypt, according to the latest data, more than half of all women give birth by CS without much difference between urban and rural areas [24]. Some possible reasons for increasing CS rates are repeatedly reported in studies from many countries such as fear of pain; concerns about genital modifications after vaginal delivery; misconception that CS is safer for the baby; the convenience for health professionals and also for the mother and family; fear of medical litigation and lower tolerance to any complications or outcomes other than the perfect baby [9, 25–29]. Other cultural factors are more country-specific. For example, in China, choosing the date of the baby’s delivery on the basis of luck and fate for the future of the baby by some people is one of the explanations for scheduling a CS [10]. On the other hand, several European countries have managed to control their CS rates over time. It is noteworthy that Finland has one of the lowest increases not only in Europe but among all countries included in this analysis (AAIR: 0.4%; from 13.5% to 14.7%). Iceland and Norway also present a very small increase and Sweden, despite the slightly higher rise, has a CS rate of 16.2% in 2011. Although there will certainly be differences in population characteristics such as the prevalence of obesity, the proportion of nulliparous or of older women or multiple births, these differences are unlikely to explain the wide variations in CS rates in European countries. Factors associated with higher rates of vaginal deliveries may include strict policies on “maternal request” CS, cultural or social pressure, differences in the legal framework for medical litigation and strategies favouring home births or a midwifery-led approach to births [30–32].

Perhaps due to the complexity of all these scenarios and the many interconnected factors that contribute to increasing CS rates, interventions tested to reduce unnecessary CS have only shown moderate success to date [33, 34]. Some case-studies have been published recently pointing to interventions such as high-quality midwifery-led unit for delivery as an effective way to reduce CS [32] and professional associations have released recommendations for the safe prevention of primary caesarean sections [35]. However, considering solely medical factors in this complex scenario is likely to be a futile effort to reduce unnecessary CS. Factors associated to women’s fears and lives and societal and cultural beliefs are very likely contributing to the increase and need to be included in the equation. High-quality research is needed in the future to explore the possible impact of these different factors when considering potential interventions to reduce unnecessary CS.

In the midst of the general worldwide rise in CS rates, it is particularly alarming to notice the lack of increase in these rates in Africa, mainly in sub-Saharan Africa where figures remain unchanged over the last decades. Broad health systems deficiencies and lack of resources continue to be limiting factors to expand access and quality care. The WHO Statement on Caesarean Section Rates published in 2015 emphasized that “Every effort should be made to provide caesarean sections to women in need, rather than striving to achieve a specific rate” [23, 36].

Strengths of the estimates presented here are the extensive and systematic approach for data searching which resulted in a high global coverage for this analysis; and the rigorous criteria used to include only nationally representative data. Nevertheless, this analysis has several limitations. Coverage was lower for the trend analysis (121 countries, 90% of the worldwide live births were included) than for the current estimates (150 countries, 96%). As expected, longitudinal data necessary to study trends were more scarce in least developed countries (coverage of 74.5%). Africa was the region with the lowest coverage (81.8% of live births). However, we do not expect the missing countries to have higher access to obstetric care and CS. In addition data for Oceania reflects mainly Australia and New Zealand. To calculate global and regional CS estimates, we used the latest national CS rate made available for any given country between 2005–2014. Data prior to 2005 were considered too old for inclusion. However, in face of the present global situation, it could be anticipated that countries with latest data in the earlier part of the range (e.g. 2005–2008) could have currently higher CS rates than those used in the analysis if data would have been available. For this reason, these estimates could be considered somewhat underestimates.

Another limitation of the study was the use of different sources of data for CS. For some countries we relied on estimates and reports from government national databases accounting for all the births in the country. For others, particularly in less/least developed countries, we used surveys which sampled a proportion of women in the country and whose timeframe (to which the data refer) is also different. In order to minimize distortion from different sources, for each country we used only one type of source. Despite its limitations, this multi-source approach to collecting nationally-representative estimates is widely used and accepted internationally [37, 38].

Global and regional estimates, as presented here, give a summary overview of the world and each region but they conceal important differences between countries. A striking example is the inequity in the use of CS between Northern and sub-Saharan Africa (Fig 2). Moreover, although estimates at country level are useful for governments and policy-makers to assess overall progress in maternal and infant health and to plan emergency obstetric care and resource utilization [39], they are merely averages and conceal important inequalities within countries [12]. In many cases, these averages are the result of low CS rates in the poorest segments of the population and higher, probably medically unjustified, use in the richest segments [12]. Ideally, CS rates should be monitored at subnational and hospital levels in a standardized and action-oriented manner with the inclusion of maternal and perinatal outcomes to assess optimal CS rates [23, 36]. WHO proposed in 2015 the use of the Robson Classification system as a global standard for assessing, monitoring and comparing CS rates [23, 36] and its use has increased spontaneously worldwide over the last decade [40, 41]. This classification allows analyses of CS rates according to important maternal and fetal variables (e.g. parity, previous CS delivery, onset of labour, gestational age, number of foetuses and presentation) which can help to understand differences in obstetric populations (case-mix) and CS rates per groups between facilities or regions and over time. As many countries are currently adopting the Robson classification at national level, we hope to perform more detailed analyses about changes in CS rates in future updates.

Current rates of CS, except for the least developed countries, are consistently higher than what is considered medically justifiable [23, 42]. The scientific, public health and medical community have raised concern about this global epidemic while the search for ideas and interventions to reduce unnecessary CS is on-going [33, 34]. However, the rational and responsible reduction of unnecessary CS is not a trivial task and it will take considerable time and efforts. Monitoring both CS rates and outcomes is essential to ensure that policies, practices and actions for the optimization of the utilization of CS lead to improved maternal and infant outcomes.

## Supporting Information

S1 File Data and sources. First and latest available CS rate data points per country, the year, total number of data points used for this analysis and sources of the data.
(PDF)

S2 File Data on caesarean section rates. Data set compiled for the analysis of caesarean section rates and trends.
(PDF)

## References

[1] Gregory KD, Jackson S, Korst L, Fridman M. Cesarean versus vaginal delivery: whose risks? Whose benefits? Am J Perinatol. 2012;29(1):7–18. 10.1055/s-0031-1285829 21833896

[2] Huang X, Lei J, Tan H, Walker M, Zhou J, Wen SW. Cesarean delivery for first pregnancy and neonatal morbidity and mortality in second pregnancy. Eur J Obstet Gynecol Reprod Biol. 2011;158(2):204–8. 10.1016/j.ejogrb.2011.05.006 21641102

[3] Timor-Tritsch IE, Monteagudo A. Unforeseen consequences of the increasing rate of cesarean deliveries: early placenta accreta and cesarean scar pregnancy. A review. Am J Obstet Gynecol. 2012;207(1):14–29. 10.1016/j.ajog.2012.03.007 22516620

[4] Marshall NE, Fu R, Guise JM. Impact of multiple cesarean deliveries on maternal morbidity: a systematic review. Am J Obstet Gynecol. 2011;205(3):262 e1-8. 10.1016/j.ajog.2011.06.035 22071057

[5] Lumbiganon P, Laopaiboon M, Gulmezoglu AM, Souza JP, Taneepanichskul S, Ruyan P, et al Method of delivery and pregnancy outcomes in Asia: the WHO global survey on maternal and perinatal health 2007–08. Lancet. 2010;375(9713):490–9. 10.1016/S0140-6736(09)61870-5 20071021

[6] Souza JP, Gulmezoglu A, Lumbiganon P, Laopaiboon M, Carroli G, Fawole B, et al Caesarean section without medical indications is associated with an increased risk of adverse short-term maternal outcomes: the 2004–2008 WHO Global Survey on Maternal and Perinatal Health. BMC medicine. 2010;8:71 10.1186/1741-7015-8-71 21067593 PMC2993644

[7] Lin HC, Xirasagar S. Institutional factors in cesarean delivery rates: policy and research implications. Obstet Gynecol. 2004;103(1):128–36. 14704256 10.1097/01.AOG.0000102935.91389.53

[8] Linton A, Peterson MR, Williams TV. Effects of maternal characteristics on cesarean delivery rates among U.S. Department of Defense healthcare beneficiaries, 1996–2002. Birth. 2004;31(1):3–11. 15015987 10.1111/j.0730-7659.2004.0268.x

[9] Zwecker P, Azoulay L, Abenhaim HA. Effect of fear of litigation on obstetric care: a nationwide analysis on obstetric practice. Am J Perinatol. 2011;28(4):277–84. 10.1055/s-0030-1271213 21249618

[10] Mi J, Liu F. Rate of caesarean section is alarming in China. Lancet. 2014;383(9927):1463–4. 10.1016/S0140-6736(14)60716-9 24766963

[11] Gibbons L, Belizan JM, Lauer JA, Betran AP, Merialdi M, Althabe F. Inequities in the use of cesarean section deliveries in the world. Am J Obstet Gynecol. 2012;206(4):331 e1-19. 10.1016/j.ajog.2012.02.026 22464076

[12] Ronsmans C, Holtz S, Stanton C. Socioeconomic differentials in caesarean rates in developing countries: a retrospective analysis. Lancet. 2006;368(9546):1516–23. 17071285 10.1016/S0140-6736(06)69639-6

[13] Betran AP, Merialdi M, Lauer JA, Bing-shun W, Thomas J, Van Look P, et al Rates of caesarean section: analysis of global, regional and national estimates. Paediatric and Perinatal Epidemiology. 2007;21:98–113. 17302638 10.1111/j.1365-3016.2007.00786.x

[14] The DHS Program—Demographic and Health Surveys: USAID; [cited 2015 23 March 2015]. Available: http://dhsprogram.com/. Accessed 23 March 2015.

[15] Multiple Indicator Cluster Surveys: UNICEF. Available: http://mics.unicef.org/.

[16] Stanton CK, Dubourg D, De Brouwere V, Pujades M, Ronsmans C. Reliability of data on caesarean sections in developing countries. Bull World Health Organ. 2005;83(6):449–55. 15976896 PMC2626266

[17] European Health for All database: World Health Organization—Regional Office for Europe; [cited 2015 23 March 2015]. Available: http://www.euro.who.int/en/data-and-evidence/databases/european-health-for-all-database-hfa-db. Accessed 23 March 2015.

[18] Composition of macro geographical (continental) regions, geographical sub-regions, and selected economic and other grouping: United Nationas Statistics Division; [cited 2015 23 March 2015]. Available from: http://unstats.un.org/unsd/methods/m49/m49regin.htm. Accessed 23 March 2015.

[19] United Nations. World Population prospects. The 2012 revision New York, USA: United Nations; 2013 [cited 2013]. Available: http://esa.un.org/wpp/unpp/panel_population.htm. Accessed 2013.

[20] Bruggmann D, Lohlein LK, Louwen F, Quarcoo D, Jaque J, Klingelhofer D, et al Caesarean Section-A Density-Equalizing Mapping Study to Depict Its Global Research Architecture. International journal of environmental research and public health. 2015;12(11):14690–708. 10.3390/ijerph121114690 26593932 PMC4661674

[21] Vogel JP, Betran AP, Vindevoghel N, Souza JP, Torloni MR, Zhang J, et al Use of the Robson classification to assess caesarean section trends in 21 countries: a secondary analysis of two WHO multicountry surveys. The Lancet Global health. 2015;3(5):e260–70. 10.1016/S2214-109X(15)70094-X 25866355

[22] Victora CG, Barros FC. Beware: unnecessary caesarean sections may be hazardous. Lancet. 2006;367(9525):1796–7. 16753467 10.1016/S0140-6736(06)68780-1

[23] WHO Statement on Caesarean Section Rates. Geneva: World Health Organization; 2015 (WHO/RHR/15.02).

[24] Ministry of Health and Population [Egypt], El-Zanaty Associates [Egypt], ICF International. The 2014 Egypt Demographic and Health Survey (2014 EDHS). Main Findings. Cairo, Egypt 2015.

[25] Hellerstein S, Feldman S, Duan T. China's 50% caesarean delivery rate: is it too high? BJOG. 2015;122(2):160–4. 10.1111/1471-0528.12971 25138909

[26] Abdel-Aleem H, Shaaban OM, Hassanin AI, Ibraheem AA. Analysis of cesarean delivery at Assiut University Hospital using the Ten Group Classification System. Int J Gynaecol Obstet. 2013;123(2):119–23. 10.1016/j.ijgo.2013.05.011 23958586

[27] Torloni MR, Betran AP, Montilla P, Scolaro E, Seuc A, Mazzoni A, et al Do Italian women prefer cesarean section? Results from a survey on mode of delivery preferences. BMC Pregnancy Childbirth. 2013;13:78 10.1186/1471-2393-13-78 23530472 PMC3621281

[28] Angeja AC, Washington AE, Vargas JE, Gomez R, Rojas I, Caughey AB. Chilean women's preferences regarding mode of delivery: which do they prefer and why? BJOG. 2006;113(11):1253–8. 17014679 10.1111/j.1471-0528.2006.01069.x

[29] Torloni MR, Daher S, Betran AP, Widmer M, Souza JP, Montilla P, et al Portrayal of caesarean section in Brazilian women's magazines: a 20 year review. BMJ. 2011;342:d276 10.1136/bmj.d276 21266421 PMC3026601

[30] Raisanen S, Gissler M, Kramer MR, Heinonen S. Influence of delivery characteristics and socioeconomic status on giving birth by caesarean section—a cross sectional study during 2000–2010 in Finland. BMC Pregnancy and Childbirth. 2014;14(1). 10.1186/1471-2393-14-120 PMC3999387 24678806

[31] Macfarlane A, Blondel B, Mohangoo A, Cuttini M, Nijhuis J, Novak Z, et al Wide differences in mode of delivery within Europe: risk-stratified analyses of aggregated routine data from the Euro-Peristat study. BJOG. 2015. [Epub ahead of print]. 10.1111/1471-0528.13284 25753683

[32] Renfrew MJ, McFadden A, Bastos MH, Campbell J, Channon AA, Cheung NF, et al Midwifery and quality care: findings from a new evidence-informed framework for maternal and newborn care. Lancet. 2014;384(9948):1129–45. 10.1016/S0140-6736(14)60789-3 24965816

[33] Khunpradit S, Tavender E, Lumbiganon P, Laopaiboon M, Wasiak J, Gruen RL. Non-clinical interventions for reducing unnecessary caesarean section. Cochrane Database Syst Rev. 2011;(6):CD005528 10.1002/14651858.CD005528.pub2 21678348

[34] Hartmann KE, Andrews JC, Jerome RN, Lewis RM, Likis FE, Nikki McKoy J, et al Strategies To Reduce Cesarean Birth in Low-Risk Women Comparative Effectiveness Review. Rockville, MD, United States: Agency for Healthcare Research and Quality; 2012. 23367527

[35] American College of Obstetricians and Gynecologist, Society for Maternal-Fetal Medicine. Obstetric Care Consensus. Safe Prevention of the Primary Cesarean Delivery. 2014.

[36] Betrán AP, Torloni MR, Zhang J, Gülmezoglu AM for the WHO Working Group on Caesarean Section. WHO Statement on caesarean section rates: a commentary. BJOG. 2015. [Epub ahead of print].

[37] Betran AP, Torloni MR, Zhang J, Ye J, Mikolajczyk R, Deneux-Tharaux C, et al What is the optimal rate of caesarean section at population level? A systematic review of ecologic studies. Reprod Health. 2015;12:57 10.1186/s12978-015-0043-6 26093498 PMC4496821

[38] Stevens GA, Finucane MM, De-Regil LM, Paciorek CJ, Flaxman SR, Branca F, et al Global, regional, and national trends in haemoglobin concentration and prevalence of total and severe anaemia in children and pregnant and non-pregnant women for 1995–2011: a systematic analysis of population-representative data. The Lancet Global health. 2013;1(1):e16–25. 10.1016/S2214-109X(13)70001-9 25103581 PMC4547326

[39] Monitoring emergency obstetric care: a handbook. Geneva, Switzerland: World Health Organization, 2009.

[40] Torloni MR, Betran AP, Souza JP, Widmer M, Allen T, Gulmezoglu M, et al Classifications for cesarean section: a systematic review. PLoS ONE. 2011;6(1):e14566 10.1371/journal.pone.0014566 21283801 PMC3024323

[41] Betran AP, Vindevoghel N, Souza JP, Gulmezoglu AM, Torloni MR. A Systematic Review of the Robson Classification for Caesarean Section: What Works, Doesn't Work and How to Improve It. PLoS One. 2014;9(6):e97769 10.1371/journal.pone.0097769 24892928 PMC4043665

[42] Appropriate technology for birth. Lancet. 1985;2(8452):436–7. 2863457

