# Supplementary material for: The Increasing Trend in Caesarean Section Rates: Global, Regional and National Estimates: 1990-2014
Source: PLoS One. 2016 Feb 5;11(2):e0148343. doi: 10.1371/journal.pone.0148343 (PMC4743929; doi:10.1371/journal.pone.0148343)
Supplement: S1 File — First and latest available CS rate data points per country, the year, total number of data points used for this analysis and sources of the data. (PDF) [file pone.0148343.s001.pdf]

Supporting information: S1 File. Data and sources. First and last caesarean section rate and sources. Number of data points included in the longitudinal analysis.

Country	Cross sectional analysis (n=150)	Trend analysis (n=121)	First year	First CS (%)	Last year	Last CS (%)	Data points (N)	SOURCES
Afghanistan	Yes	No	-	-	2011	3.6	1	Central Statistics Organisation (CSO) and UNICEF. Afghanistan Multiple Indicator Cluster Survey 2010-2011: Final Report. Kabul: Central Statistics Organisation (CSO) and UNICEF; 2012.
Albania	Yes	Yes	1992	9.3	2011	30	20	European Health for All Database (HFA-DB) [online database updated on April 2014]. WHO Regional Office for Europe; 2014 (http://data.euro.who.int/hfadb/, accessed 15 April 2015).
Algeria	Yes	Yes	1992	6.3	2012	16.3	2	• Ministère de la Santé, de la Population, Office National des Statistiques, Ligue des Etats Arab. Enquête Algerienne sur la Santé de la mere et de l'enfant. Rapport principal. Algerie; 1993.<br>• Ministère de la Santé, de la Population et de la Réforme Hospitalière, Office National des Statistiques. Algerie Enquête Nationale à Indicateurs Multiples. Rapport Principal. Algerie; 2013.
Argentina	Yes	No	-	-	2011	29.1	1	Ariel Karolinski, Raúl Mercer, Pablo Salgado, Celina Ocampo y otros. Primer informe nacional de relevamiento epidemiológico del SIPGestión: desarrollo e implementación a escala nacional de un sistema de información en salud de la mujer y perinatal en Argentina. Buenos Aires: Organización Panamericana de la Salud; Ministerio de Salud de la Nación; 2013.
Armenia	Yes	Yes	1990	3.4	2012	22.5	23	European Health for All Database (HFA-DB) [online database updated on April 2014]. WHO Regional Office for Europe; 2014 (http://data.euro.who.int/hfadb/, accessed 15 April 2015).
Australia	Yes	Yes	1991	18.0	2012	32.4	22	• Lancaster P, Huang J, Pedisich E. Australia's mothers and babies 1991. Perinatal Statistics Series No. 1. AIHW National Perinatal Statistics Unit: Sydney, Australia. 1994.<br>• Lancaster P, Huang J, Pedisich E. Australia's mothers and babies 1992. Perinatal Statistics Series No. 2. AIHW National Perinatal Statistics Unit: Sydney, Australia. 1995.<br>• Lancaster P, Huang J. Lin M. Australia's mothers and babies 1993. Perinatal Statistics Series No. 3. AIHW National Perinatal Statistics Unit: Sydney, Australia, 1996.<br>• Day P, Lancaster P, Huang J. Australia's mothers and babies 1995. Perinatal

Country	Cross sectional analysis (n=150)	Trend analysis (n=121)	First year	First CS (%)	Last year	Last CS (%)	Data points (N)	SOURCES
								Statistics Series No. 6. AIHW National Perinatal Statistics Unit: Sydney, Australia. 1997.<br>• Day P, Sullivan EA, Lancaster P. Australia's mothers and babies 1996. Perinatal Statistics Series No. 7. AIHW Cat. No. PER 4. Sydney, Australia: Australian Institute of Health and Welfare National Perinatal Statistics Unit, 1999.<br>• Day P, Sullivan EA, Ford J, Lancaster P. Australia's mothers and babies 1997. Perinatal Statistics Series no.9. AIHW Cat. No. PER 12. Sydney, Australia: AIHW National Perinatal Statistics Unit, 1999.<br>• Nassar N, Sullivan EA, Lancaster P, Day P. Australia's mothers and babies 1998. Perinatal Statistics Series no. 10 AIHW Cat. No. PER 15. Sydney, Australia: AIHW National Perinatal Statistics Unit, 2000.<br>• Nassar N, Sullivan EA. Australia's mothers and babies 1999. Perinatal Statistics Series no. 11. AIHW Cat. No. PER 19. Sydney, Australia: AIHW National Perinatal Statistics Unit, 2001.<br>• AIHW NPSU. Australia's mothers and babies 2000. Perinatal Statistics Series no. 12. AIHW Cat. No. PER 21. Canberra, Australia: AIHW National Perinatal Statistics Unit, 2003.<br>• Laws PJ, Sullivan EA. Australia's mothers and babies 2001. Perinatal Statistics Series No. 13. AIHW Cat. No. PER 25. Sydney, Australia: AIHW National Perinatal Statistics Unit, 2004.<br>• Laws PJ, Sullivan EA. Australia's mothers and babies 2002. Perinatal Statistics Series No. 15. AIHW Cat. No. PER 28. Sydney, Australia: AIHW National Perinatal Statistics Unit, 2004.<br>• Laws PJ, Sullivan EA. Australia's mothers and babies 2003. Perinatal Statistics Series No. 16. AIHW Cat. No. PER 29. Sydney, Australia: AIHW National Perinatal Statistics Unit, 2005.<br>• Laws PJ, Grayson N, Sullivan EA. Australia's mothers and babies 2004. Perinatal statistics series no. 18. AIHW cat. no. PER 34. Sydney, Australia: AIHW National Perinatal Statistics Unit, 2006.<br>• Laws PJ, Abeywardana S, Walker J, Sullivan EA. Australia's mothers and babies 2005. Perinatal statistics series no. 20. Cat. no. PER 40. Sydney,

Country	Cross sectional analysis (n=150)	Trend analysis (n=121)	First year	First CS (%)	Last year	Last CS (%)	Data points (N)	SOURCES
								Australia: AIHW National Perinatal Statistics Unit, 2007.<br>• Laws PJ, Hilder L. Australia's mothers and babies 2006. Perinatal statistics series no. 22. Cat. no. PER 46. Sydney, Austalia: AIHW National Perinatal Statistics Unit, 2008.<br>• Laws P, Sullivan EA. Australia's mothers and babies 2007. Perinatal statistics series no. 23. Cat. no. PER 48. Sydney, Austalia: AIHW National Perinatal Statistics Unit, 2009<br>• Laws PJ, Li Z, Sullivan EA. Australia's mothers and babies 2008. Perinatal statistics series no. 24. Cat. No. PER 50. Canberra, Australia, 2010<br>• Li Z, McNally L, Hilder L, Sullivan EA. Australia's mothers and babies 2009. Perinatal statistics series no. 25. Cat. no. PER 52. Sydney: AIHW National. Perinatal Epidemiology and Statistics Unit, 2011.<br>• Li Z, Zeki R, Hilder L and Sullivan EA. Australia's mothers and babies 2010. Perinatal statistics series no. 27. Cat. no. PER 57. Canberra: AIHW National Perinatal Epidemiology and Statistics Unit, 2012.<br>• Li Z, Zeki R, Hilder L & Sullivan EA. Australia's mothers and babies 2011. Perinatal statistics series no. 28. Cat. no. PER 59. Canberra: AIHW National Perinatal Epidemiology and Statistics Unit, 2013.<br>• Hilder L, Zhichao Z, Parker M, Jahan S, Chambers GM. Australia's mothers and babies 2012. Perinatal statistics series no. 30. Cat. no. PER 69. Canberra: AIHW; 2014.
Austria	Yes	Yes	1995	12.4	2011	28.3	17	European Health for All Database (HFA-DB) [online database updated on April 2014]. WHO Regional Office for Europe; 2014 (http://data.euro.who.int/hfadb/ , accessed 15 April 2015).
Azerbaijan	Yes	Yes	1990	1.4	2012	16.7	23	European Health for All Database (HFA-DB) [online database updated on April 2014]. WHO Regional Office for Europe; 2014 (http://data.euro.who.int/hfadb/ , accessed 15 April 2015).
Bahrain	Yes	Yes	1995	16	2012	26	10	• Ministry of Health. Health Statistics of 2008. Bahrain, 2009.<br>• Ministry of Health. Health Statistics of 2010. Bahrain, 2011.<br>• Tawfeeq N, Farid SM. Bahrain Family Health Survey 1995. Principal Report. Manama, Bahrain: Ministry of Health; 2000.<br>• Ministry of Health. Health Statistics of 2012. Bahrain; 2013. Accessed on 16

Country	Cross sectional analysis (n=150)	Trend analysis (n=121)	First year	First CS (%)	Last year	Last CS (%)	Data points (N)	SOURCES
								October 2014 (http://www.moh.gov.bh/PDF/Publications/statistics/HS2012/hs2012_e.htm).
Bangladesh	Yes	Yes	1999	2.4	2013	19.1	5	• National Institute of Population Research and Training (NIPORT), Mitra and Associates (MA), and ORC Macro (ORCM). Bangladesh Demographic and Health Survey 1999-2000. Dhaka, Bangladesh and Calverton; 2001.<br>• National Institute of Population Research and Training (NIPORT), Mitra and Associates, and ORC Macro. Bangladesh Demographic and Health Survey 2004. Dhaka, Bangladesh and Calverton, Maryland [USA]: National Institute of Population Research and Training, Mitra and Associates, and ORC Macro; 2005.<br>• National Institute of Population Research and Training (NIPORT), Mitra and Associates, and Macro International. Bangladesh Demographic and Health Survey 2007. Dhaka, Bangladesh and Calverton, Maryland, USA: National Institute of Population Research and Training, Mitra and Associates, and Macro International; 2009.<br>• National Institute of Population Research and Training (NIPORT), Mitra and Associates, and ICF International. Bangladesh Demographic and Health Survey 2011. Dhaka, Bangladesh and Calverton, Maryland, USA: NIPORT, Mitra and Associates, and ICF International; 2013.<br>• Maryland [USA]: National Institute of Population Research and Training, Mitra and Associates, and ORC Macro.Progotir Pathey Multiple Indicators Custer Survey 2012-2013, Key Findings. Bangladesh Bureau of Statistics and UNICEF Bangladesh. Dhaka, Bangladesh; 2014.
Belarus	Yes	Yes	1993	9.2	2012	25.3	20	European Health for All Database (HFA-DB) [online database updated on April 2014]. WHO Regional Office for Europe; 2014 (http://data.euro.who.int/hfadb/, accessed 15 April 2015).
Belgium	Yes	Yes	1990	10.4	2010	19.7	11	European Health for All Database (HFA-DB) [online database updated on April 2014]. WHO Regional Office for Europe; 2014 (http://data.euro.who.int/hfadb/, accessed 15 April 2015).

Country	Cross sectional analysis (n=150)	Trend analysis (n=121)	First year	First CS (%)	Last year	Last CS (%)	Data points (N)	SOURCES
Belize	Yes	Yes	1991	8.4	2011	28.9	3	Statistical Institute of Belize (SIB) and UNICEF. The Belize Multiple Indicator Cluster Survey (MICS) 2011. SIB and UNICEF: Belize; 2012.
Benin	Yes	Yes	1996	2.2	2011	5.4	4	• Kodjogbé, Nicalse, Gora Mboup, Justin Tossou, IAopoldine de Souza, Timothée Gandaho, Alphonse Guédémé, Thomas Houedokoho, Rafatou Houndékon, Thomas Tohouegnon, Suzanne Zomahoun, Virgile Capo-Chichi, et Andrée Cossi. Enquête Démographique et de Santé, République de Bénin 1996. Calverton, Maryland USA : Institut National de la Statistique et de l'Analyse Économique et Macro International Inc; 1997.<br>• Institut National de la Statistique et de l'Analyse Économique (INSAE) et ORC Macro. Enquête Démographique et de Santé au Bénin 2001. Calverton, Maryland, USA : Institut National de la Statistique et de l'Analyse Économique et ORC Macro; 2002.<br>• Institut National de la Statistique et de l'Analyse Économique (INSAE) [Bénin] et Macro International Inc.: Enquête Démographique et de Santé (EDSB-III) - Bénin 2006. Calverton, Maryland, USA : Institut National de la Statistique et de l'Analyse Économique et Macro International Inc; 2007.<br>• Institut National de la Statistique et de l'Analyse économique (INSAE) et ICF International. Enquête Démographique et de Santé du Bénin 2011-2012. Calverton, Maryland, USA : INSAE et ICF International; 2013.
Bhutan	Yes	No	-	-	2010	12.4	1	National Statistics Bureau. Bhutan Multiple Indicator Survey 2010.Thrimphu, Bhutan: National Statistics Bureau; 2011.
Bolivia	Yes	Yes	1989	7.9	2008	18.6	5	• Demographic Health and Surveys (DHS). Maternal and Child Health in Bolivia.. Report on the In-depth DHS Survey in Bolivia 1989. Institute for Resource Development/Macro Systems, Inc. Columbia, Maryland, USA; 1991.<br>• Instituto Nacional de Estadística, Demographic and Health Surveys. Bolivia Encuesta Nacional de Demografía y Salud 1994. Instituto Nacional de Estadística, Demographic and Health Surveys, Macro International Inc. Calverton, Maryland, USA; 1994.<br>• Instituto Nacional de Estadística, Demographic and Health Surveys. Bolivia Encuesta Nacional de Demografía y Salud 1998. Instituto Nacional de

Country	Cross sectional analysis (n=150)	Trend analysis (n=121)	First year	First CS (%)	Last year	Last CS (%)	Data points (N)	SOURCES
								Estadística, Demographic and Health Surveys, Macro International Inc. Calverton, Maryland, USA; 1998.<br>• Instituto Nacional de Estadística, Demographic and Health Surveys, Ministerio de Sálud y Deportes. Bolivia Encuesta Nacional de Demografía y Salud 2003. Instituto Nacional de Estadística, Demographic and Health Surveys, Macro International Inc. Calverton, Maryland, USA; 2004.<br>• Demographic Health and Surveys (DHS). Bolivia Encuesta Nacional de Demografía y Salud 2008. Calverton, Maryland, USA. Macro International Inc.; 2008.
Bosnia and Herzegovina	Yes	No	-	-	2012	13.9	1	The Agency for Statistics of Bosnia and Herzegovina, the Federal Ministry of Health, the Ministry of Health and Social Welfare of the Republic of Srpska and the Institute for Public Health of the Federation of Bosnia and Herzegovina. Bosnia and Herzegovina Multiple Indicator Cluster Survey (MICS) 2011–2012, Final Report. Sarajevo: UNICEF; 2013.
Brazil	Yes	Yes	1994	37.8	2012	55.6	18	MS/SVS/DASIS - Sistema de Informações sobre Nacidos Vivos - SINASC, Brasil. (http://tabnet.datasus.gov.br/cgi/deftohtm.exe?sinasc/cnv/nvuf.def) (database accessed 12 December 2014).
Bulgaria	Yes	Yes	1990	7.3	2011	33.1	22	European Health for All Database (HFA-DB) [online database updated on April 2014]. WHO Regional Office for Europe; 2014 (http://data.euro.who.int/hfadb/ , accessed 15 April 2015).
Burkina Faso	Yes	Yes	1993	1.3	2010	1.9	4	• Institut National de la Statistique et de la Démographie, Macro International Inc. Enquête Démographique et de Santé Burkina Faso 1993. Calverton, Maryland USA: Macro International Inc.; 1994.<br>• Institut National de la Statistique et de la Démographie, et Macro International Inc. Enquête Démographique et de Santé, Burkina Faso 1998-1999. Calverton, Maryland, USA: Macro International Inc.; 2000.<br>• Institut National de la Statistique et de la Démographie (INSD) et ORC Macro. Enquête Démographique et de Santé du Burkina Faso 2003. Calverton, Maryland, USA: INSD et ORC Macro; 2004.<br>• Institut National de la Statistique et de la Démographie (INSD) et ICF International. Enquête Démographique et de Santé et à Indicateurs Multiples du Burkina Faso 2010. Calverton, Maryland, USA: INSD et ICF

Country	Cross sectional analysis (n=150)	Trend analysis (n=121)	First year	First CS (%)	Last year	Last CS (%)	Data points (N)	SOURCES
								International; 2012.
Burundi	Yes	No	-	-	2010	4	1	Institut de Statistiques et d'Études Économiques du Burundi (ISTEEBU), Ministère de la Santé Publique et de la Lutte contre le Sida [Burundi] (MSPLS), et ICF International. Enquête Démographique et de Santé Burundi 2010. Bujumbura, Burundi : ISTEEBU, MSPLS, et ICF International; 2012.
Côte d'Ivoire	Yes	Yes	1994	1.8	2011	2.7	4	• Institut National de la Statistique, Ministère Délégué Auprès du Premier Ministre, Chargé de l'Economie, des Finances et du Plan, Macro International Inc. Enquête Démographique et de Santé 1994. Calverton, Maryland, USA: Macro International Inc.; 1995.<br>• Institut National de la Statistique [Côte d'Ivoire] et ORC Macro. Enquête Démographique et de Santé, Côte d'Ivoire 1998-1999. Calverton, Maryland USA : Institut National de la Statistique et ORC Macro; 2001.<br>• Institut National de la Statistique (INS) et Ministère de la Lutte contre le Sida [Côte d'Ivoire] et ORC Macro. Enquête sur les Indicateurs du Sida, Côte d'Ivoire 2005. Calverton, Maryland, U.S.A. : INS et ORC; 2006.<br>• Macro Institut National de la Statistique (INS) et ICF International. Enquête Démographique et de Santé et à Indicateurs Multiples de Côte d'Ivoire 2011-2012. Calverton, Maryland, USA : INS et ICF International; 2012.
Cambodia	Yes	Yes	2000	0.8	2010	3	3	• National Institute of Statistics, Directorate General for Health [Cambodia], and ORC Macro. Cambodia Demographic and Health Survey 2000. Phnom Penh, Cambodia, and Calverton, Maryland USA: National Institute of Statistics, Directorate General for Health, and ORC Macro, 2001.<br>• National Institute of Public Health, National Institute of Statistics [Cambodia] and ORC Macro. Cambodia Demographic and Health Survey 2005. Phnom Penh, Cambodia and Calverton, Maryland, USA: National Institute of Public Health, National Institute of Statistics and ORC Macro, 2006.<br>• National Institute of Statistics, Directorate General for Health, and ICF

Country	Cross sectional analysis (n=150)	Trend analysis (n=121)	First year	First CS (%)	Last year	Last CS (%)	Data points (N)	SOURCES
								Macro. Cambodia Demographic and Health Survey 2010. Phnom Penh, Cambodia and Calverton, Maryland, USA: National Institute of Statistics, Directorate General for Health, and ICF Macro; 2011.
Cameroon	Yes	Yes	1991	2.4	2011	3.8	4	• Direction Nationale du Deuxième Recensement Général de la Population et de l'Habitat, Macro International Inc. Enquête Démographique et de Santé du Cameroun 1991. Calverton, Columbia, Maryland, USA: Macro International Inc.; 1992.<br>• Fotso, Médard, René Ndonou, Paul Roger Libité, Martin Tsafack, Roger Wakou, Aboubakar Ghapoutsa, Samuel Kamga, Pierre Kemgo, Michel Kwekem Fankam, Antoine Kamdoum, Bernard Barrère. Enquête Démographique et de Santé, Cameroun 1998. Calverton, Maryland, USA: Bureau Central des Recensements et des Études de Population et Macro International Inc.; 1999.<br>• Institut National de la Statistique (INS) et ORC Macro. Enquête Démographique et de Santé du Cameroun 2004. Calverton, Maryland, USA: INS et ORC Macro; 2005.<br>• Institut National de la Statistique (INS) et ICF. International. Enquête Démographique et de Santé et à Indicateurs Multiples du Cameroun 2011. Calverton, Maryland, USA : INS et ICF International; 2012.
Canada	Yes	Yes	1992	18.2	2012	27.1	19	• Health Canada. Canadian Perinatal Health Report, 2000. Ottawa: Minister of Public Works and Government Services Canada, 2000.<br>• Ottawa: Minister of Public Works and Government Services Canada; 2000.<br>• Public Health Agency of Canada. Canadian Perinatal Health Report, 2008 Edition. Ottawa; 2008.<br>• Public Health Agency of Canada. Perinatal Health Indicators for Canada 2011. Ottawa; 2012.<br>• Canadian Institute for Health Information. Health Indicators 2013. Ottawa, Ontario : Canadian Institute for Health Information, ; 2014.
Central African	Yes	Yes	1994	1.9	2010	4.5	2	• Ndamobissi, Robert, Gora Mboup et Edwige Opportune Nguélébé. Enquête Démographique et de Santé, République Centrafricaine 1994-95. Calverton,

Country	Cross sectional analysis (n=150)	Trend analysis (n=121)	First year	First CS (%)	Last year	Last CS (%)	Data points (N)	SOURCES
Republic								Maryland, USA: Direction des Statistiques Démographiques et Sociales et Macro International Inc.; 1995.<br>• ICASEES. Enquête par grappes à indicateurs multiples MICS, RCA 2010 Rapport final. Bangui: RCA ICASEES; 2010.
Chad	Yes	Yes	1996	0.5	2010	1.5	3	• Ouagadjio, Bandoumal, Kostelngar Nodjimadji, Joël Nodjimbatem Ngoniri, Ningam Ngakoutou, Keumaye Ignégongba, Joël S. Tokindang, Oumdague Kouo, Bernard Barrère, et Monique Barrèere. Enquête Démographique et de Santé, Tchad 1996–97. Calverton, Maryland, USA; Bureau Central du Recensement et Macro International Inc; 1998.<br>• Ouagadjio, Bandoumal, Kostelngar Nodjimadji, Tchobkréo Bagamla, Riradjim Madnodji, Joël Sibaye Tokindang, Ningam Ngakoutou, Joël Nodjimbatem Ngoniri, Caman Bédaou, Donato Koyalta, Bernard Barrère, Monique Barrère. Enquête Démographique et de Santé Tchad 2004. Calverton, Maryland, U.S.A. : INSEED et ORC Macro; 2004.<br>• Ministère du Plan, de l'Economie et de la Coopération Internationale. Institut National de la Statistique, des études économiques et Démographiques (INSEED), United Nations Population Fund, United Nations Children's Fund. Enquête par grappes à indicateurs multiples-Rapport Final, Tchad; 2012.
China	Yes	Yes	1990	4.4	2011	36.2	20	Qian JC. Analysis on cesarean section rates in China from 2003 to 2011. Population and Development.2012,18(5):39-42.
Colombia	Yes	Yes	1990	16	2012	43.4	17	Departamento Administrativo Nacional de Estadística (DANE). Estadísticas Vitales - Nacimientos. Bogotá D.C., Colombia; 2014 (website accessed 15 January 2015. http://www.dane.gov.co/).
Comoros	Yes	Yes	1996	5.3	2012	9.6	2	• Mondoha, Kassim A., Juan Schoemaker et Monique Ban'ère. Enquête Démographique et de Santé, Comores 1996. Calverton, Maryland : Centre National de Documentation et de Recherche Scientifique et Macro International Inc.; 1997.<br>• Direction Générale de la Statistique et de la Prospective (DGSP) et ICF International.. Enquête Démographique et de Santé et à Indicateurs Multiples aux Comores 2012. Rockville, MD 20850, USA : DGSP et ICF International, 2014.
Congo	Yes	No	-	-	2011	5.8	1	Centre Nationale de la Statistique et des Études Économiques (CNSEE) [Congo]

Country	Cross sectional analysis (n=150)	Trend analysis (n=121)	First year	First CS (%)	Last year	Last CS (%)	Data points (N)	SOURCES
								et ICF International. Enquête Démographique et de Santé du Congo (EDSC-II) 2011-2012. Calverton, Maryland, USA: CNSEE et ICF International; 2013.
Costa Rica	Yes	Yes	1990	19.8	2013	21.9	24	Caja Costarricense del Seguro Social. área de Estadística en Salud 2013. San José, Costa Rica (website accessed 19 December 2014. http://www.ccss.sa.cr/estadisticas_salud_docs).
Croatia	Yes	Yes	1990	5.3	2012	20.16	22	European Health for All Database (HFA-DB) [online database updated on April 2014]. WHO Regional Office for Europe; 2014 (http://data.euro.who.int/hfadb/, accessed 15 April 2015).
Cyprus	Yes	No	2005	6.9	2010	11.4	6	European Health for All Database (HFA-DB) [online database updated on April 2014]. WHO Regional Office for Europe; 2014 (http://data.euro.who.int/hfadb/, accessed 15 April 2015).
Czech Republic	Yes	Yes	1990	7.6	2011	23.3	31	European Health for All Database (HFA-DB) [online database updated on April 2014]. WHO Regional Office for Europe; 2014 (http://data.euro.who.int/hfadb/, accessed 15 April 2015).
Dem. People's Rep. of Korea	Yes	No	-	-	2009	12.5	1	Central Bureau of Statistics, UNICEF. Multiple Indicator Cluster Survey 2009. Final Report. Democratic People's Republic of Korea; 2010.
Dem. Rep. of the Congo	Yes	No	-	-	2010	7.2	1	Institut National de la Statistique, UNICEF. Enquête par grappes a Indicateurs Multiples en Républic Démocratique du Congo - (MICS-RDC 2010) Rapport Final. Républic Démocratique du Congo; 2011.
Denmark	Yes	Yes	1990	12.6	2010	20.8	21	European Health for All Database (HFA-DB) [online database updated on April 2014]. WHO Regional Office for Europe; 2014 (http://data.euro.who.int/hfadb/, accessed 15 April 2015).
Dominican Republic	Yes	Yes	1991	20	2013	56.4	7	• Instituto de Estudios de Población y Desarrollo (IEPD), Oficina Nacional de Planificacion (ONAPLAN), IRD Macro International Inc. Republica Dominicana Encuesta Demográfica y de Salud 1991. Instituto de Estudios de Población y Desarrollo (IEPD), Oficina Nacional de Planificacion (ONAPLAN), IRD Macro International Inc. Columbia, Maryland; 1992.<br>• Centro de Estudios Sociales y Demográficos (CESDEM), Asociación Dominicana Pro Bienestar de la Familia (PROFAMILIA), Oficina Nacional de Planificacion (ONAPLAN), Macro International Inc. Republica Dominicana

Country	Cross sectional analysis (n=150)	Trend analysis (n=121)	First year	First CS (%)	Last year	Last CS (%)	Data points (N)	SOURCES
								Encuesta Demográfica y de Salud 1996. Centro de Estudios Sociales y Demográficos (CESDEM), Asociación Dominicana Pro Bienestar de la Familia (PROFAMILIA), Oficina Nacional de Planificacion (ONAPLAN), Macro International Inc. Calverton, Maryland; 1997.<br>• Centro de Estudios Sociales y Demográficos (CESDEM), Agencia para el Desarrollo Internacional, USAID, Macro International Inc. República Dominicana Encuesta Experimental de Demografía y de Salud 1999. Centro de Estudios Sociales y Demográficos (CESDEM), Agencia para el Desarrollo Internacional, USAID, Macro International Inc. Calverton, Maryland; 2001.<br>• Centro de Estudios Sociales y Demográficos (CESDEM), Secretaría de Estado de Salud Pública y Asistencia Social (SESPAS), Comisión Ejecutiva para la Reforma del Sector Salud (CERSS), Consejo Presidencial del SIDA (COPRESIDA), Agencia de los Estados Unidos para el Desarrollo Internacional (USAID), Banco Mundial/CERSS, Banco Interamericano de Desarrollo/CERSS, Macro International Inc. República Dominicana Encuesta Demográfica y de Salud ENDESA 2002. Centro de Estudios Sociales y Demográficos (CESDEM), Secretaría de Estado de Salud Pública y Asistencia Social (SESPAS), Comisión Ejecutiva para la Reforma del Sector Salud (CERSS), Consejo Presidencial del SIDA (COPRESIDA), Agencia de los Estados Unidos para el Desarrollo Internacional (USAID), Banco Mundial/CERSS, Banco Interamericano de Desarrollo/CERSS, Macro International Inc; Calverton, Maryland; 2003.<br>• Centro de Estudios Sociales y Demográficos (CESDEM) y Macro International Inc. Encuesta Demográfica y de Salud 2007. Santo Domingo, República Dominicana: CESDEM y Macro International Inc; 2008.<br>• Ministerio de Salud Pública. Dirección General de Información y Estadísticas de Salud. Anuario Estadístico de Salud 2012. República Dominicana, 2013.<br>• Centro de Estudios Sociales y Demográficos (CESDEM) y ICF International. Encuesta Demográfica y de Salud 2013. Santo Domingo, República Dominicana: CESDEM y ICF International; 2014.
Ecuador	No*	Yes	1994	17.1	2004	25.8	3	• Centro de Estudios de Población y Paternidad Responsable (CEPAR), División de Salud Reproductiva de los Centros para el Control de Enfermedades (CDC). Encuesta de Salud Materna e Infantil (ENDEMAIN-94).

Country	Cross sectional analysis (n=150)	Trend analysis (n=121)	First year	First CS (%)	Last year	Last CS (%)	Data points (N)	SOURCES
								Quito, Ecuador; 1995.<br>• Centro de Estudios de Población y Desarrollo Social (CEPAR), Centros para el Control y Prevención de Enfermedades (CDC). Encuesta Demográfica y de Salud Materna e Infantil (ENDEMAIN-99). Informe General. Quito, Ecuador; 2001.<br>• Centro de Estudios de Población y Desarrollo Social (CEPAR), Centros para el Control y Prevención de Enfermedades (CDC). Encuesta Demográfica y de Salud Materna e Infantil (ENDEMAIN-2004). Informe Final. Quito, Ecuador; 2005.
Egypt	Yes	Yes	1992	4.6	2014	51.8	6	• El-Zanaty FH, Sayed H AA, Zaky Hassan HM, Way AA. Egypt Demographic and Health Survey 1992. Calverton, Maryland [USA]: National Population Council [Egypt] and Macro International Inc.; 1993.<br>• El-Zanaty, Fatma, Enas M. Hussein, Gihan A. Shawky, Ann A. Way, and Sunita Kishor. Egypt Demographic and Health Survey 1995. Calverton, Maryland [USA]: National Population Council [Egypt] and Macro International Inc.; 1996.<br>• El-Zanaty, Fatma and Ann Way. Egypt Demographic and Health Survey 2000. Calverton, Maryland [USA]: Ministry of Health and Population [Egypt], National Population Council and ORC Macro; 2001.<br>• El-Zanaty, Fatma and Ann Way. Egypt Demographic and Health Survey 2005. Cairo, Egypt: Ministry of Health and Population, National Population Council, El-Zanaty and Associates, and ORC Macro; 2006.<br>• El-Zanaty, Fatma and Ann Way. Egypt Demographic and Health Survey 2008. Cairo, Egypt: Ministry of Health, El-Zanaty and Associates, and Macro International; 2009.<br>• Ministry of Health and Population, El-Zanaty and Associates, The DHS Program ICF International Rockville, Maryland USA. Egypt Demographic and Health Survey 2014. Main Findings. DHS Program ICF International Rockville, Maryland USA; 2014.
El Salvador	Yes	Yes	1991	13.4	2013	29.8	12	• Asociación Demográfica Salvadoreña (ADS), Division of Reproductive Health of the Centers for Disease Control and Prevention (CDC) . Encuesta Nacional de Salud Familiar de 1993 (FESAL-93). San Salvador, El Salvador; 1994.

Country	Cross sectional analysis (n=150)	Trend analysis (n=121)	First year	First CS (%)	Last year	Last CS (%)	Data points (N)	SOURCES
								• Asociación Demográfica Salvadoreña (ADS), Division of Reproductive Health of the Centers for Disease Control and Prevention (CDC) . Encuesta Nacional de Salud Familiar de 1998 (FESAL-98). Informe Final. San Salvador, El Salvador; 2000.<br>• Asociación Demográfica Salvadoreña (ADS), Division of Reproductive Health of the Centers for Disease Control and Prevention (CDC). Encuesta Nacional de Salud Familiar de 2002/03 (FESAL-2002/03). Informe Final. San Salvador, El Salvador; 2004.<br>• Ministerio de Salud. Informe de Labores 2012-2013. San Salvador, El Salvador; 2013.<br>• Ministerio de Salud. Informe de Labores 2013-2014. San Salvador, El Salvador; 2014.
Equatorial Guinea	Yes	No	-	-	2011	6.6	1	Ministerio de Sanidad y Bienestar Social, Ministerio de Economía, Planificación e Inversiones Públicas, e ICF International. Encuesta Demográfica y de Salud (EDSGE-I) 2011. Calverton, Maryland, USA; 2012.
Eritrea	Yes	Yes	1995	1.6	2010	2.8	3	• National Statistics Office [Eritrea] and Macro International Inc. Eritrea Demographic and Health Survey, 1995. Calverton, Maryland: National Statistics Office and Macro International Inc.; 1997.<br>• National Statistics and Evaluation Office (NSEO) [Eritrea] and ORC Macro. Eritrea Demographic and Health Survey 2002. Calverton, Maryland, USA: National Statistics and Evaluation Office and ORC Macro; 2003.<br>• National Statistics Office (NSO) [Eritrea], and Fafo AIS. Eritrea Population and Health Survey 2010. Asmara, Eritrea: National Statistics Office, and Fafo Institute doer Appled International Studies; 2013.
Estonia	Yes	Yes	1990	6.2	2011	20.2	22	European Health for All Database (HFA-DB) [online database updated on April 2014]. WHO Regional Office for Europe; 2014 (http://data.euro.who.int/hfadb/ , accessed 15 April 2015).
Ethiopia	Yes	Yes	2000	0.6	2011	1.5	3	• Central Statistical Authority [Ethiopia] and ORC Macro. Ethiopia Demographic and Health Survey 2000. Addis Ababa, Ethiopia and Calverton, Maryland, USA: Central Statistical Authority and ORC Macro; 2001.<br>• Central Statistical Agency [Ethiopia] and ORC Macro. Ethiopia Demographic

Country	Cross sectional analysis (n=150)	Trend analysis (n=121)	First year	First CS (%)	Last year	Last CS (%)	Data points (N)	SOURCES
								and Health Survey 2005. Addis Ababa, Ethiopia and Calverton, Maryland, USA: Central Statistical Agency and ORC Macro; 2006.<br>• Central Statistical Agency [Ethiopia] and ICF International. Ethiopia Demographic and Health Survey 2011. Addis Ababa, Ethiopia and Calverton, Maryland, USA: Central Statistical Agency and ICF International; 2012.
Finland	Yes	Yes	1990	13.5	2011	14.7	22	European Health for All Database (HFA-DB) [online database updated on April 2014]. WHO Regional Office for Europe; 2014 (http://data.euro.who.int/hfadb/, accessed 15 April 2015).
France	Yes	Yes	1997	16.3	2011	21	15	European Health for All Database (HFA-DB) [online database updated on April 2014]. WHO Regional Office for Europe; 2014 (http://data.euro.who.int/hfadb/, accessed 15 April 2015).
Gabon	Yes	Yes	2000	5.6	2012	10	2	• Direction Générale de la Statistique et des Études Économiques (DGSEE) [Gabon] et ORC Macro. Enquête Démographique et de Santé Gabon 2000. Calverton, Maryland : Direction Générale de la Satistique et des Études Économiques, et Fonds des Nations Unies pour la Populations, et ORC Macro; 2001.<br>• Direction Générale de la Statistique (DGS) et ICF International. Enquête Démographique et de Santé du Gabon 2012. Calverton, Maryland, et Libreville, Gabon : DGS et ICF International; 2013.
Gambia	Yes	No	-	-	2010	2.5	1	The Gambia Bureau of Statistics (GBOS). The Gambia Multiple Indicator Cluster Survey 2010, Final Report. Banjul, The Gambia: The Gambia Bureau of Statistics (GBOS); 2012.
Georgia	Yes	Yes	1990	3.8	2012	36.7	23	European Health for All Database (HFA-DB) [online database updated on April 2014]. WHO Regional Office for Europe; 2014 (http://data.euro.who.int/hfadb/, accessed 15 April 2015).
Germany	Yes	Yes	1990	15.7	2009	30.3	20	European Health for All Database (HFA-DB) [online database updated on April 2014]. WHO Regional Office for Europe; 2014 (http://data.euro.who.int/hfadb/, accessed 15 April 2015).
Ghana	Yes	Yes	1993	4.4	2011	11.4	5	• Ghana Statistical Service (GSS) and Macro International Inc. (MI). Ghana Demographic and Health Survey 1993. Calverton, Maryland: GSS and MI; 1994.<br>• Ghana Statistical Service (GSS) and Macro International Inc. (MI). Ghana

Country	Cross sectional analysis (n=150)	Trend analysis (n=121)	First year	First CS (%)	Last year	Last CS (%)	Data points (N)	SOURCES
								Demographic and Health Survey 1998. Calverton, Maryland: GSS and MI; 1999.<br>• Ghana Statistical Service (GSS), Noguchi Memorial Institute for Medical Research (NMIMR), and ORC Macro. Ghana Demographic and Health Survey 2003. Calverton, Maryland: GSS, NMIMR, and ORC Macro; 2004.<br>• Ghana Statistical Service (GSS), Ghana Health Service (GHS), and ICF Macro. Ghana Demographic and Health Survey 2008. Accra, Ghana: GSS, GHS, and ICF Macro; 2009.<br>• Ghana Statistical Service. Ghana Multiple Indicator Cluster Survey with an Enhanced Malaria Module and Biomarker, 2011, Final Report. Accra, Ghana; 2011.
Guatemala	Yes	Yes	1995	8.2	2008	16.3	4	• Instituto Nacional de Estadística (INE), Macro International Inc. Guatemala Encuesta Nacional de Salud Materno Infantil 1995. Calverton, Maryland, USA: Macro International Inc.; 1996.<br>• Instituto Nacional de Estadística (INE), Macro International Inc. Guatemala Encuesta Nacional de Salud Materno Infantil 1998-1999. Calverton, Maryland, USA: Macro International Inc.; 1999.<br>• Ministerio de Salud Pública y Asistencia Social (MSPAS), Instituto Nacional de Estadística (INE). Guatemala Encuesta Nacional de Salud Materno Infantil 2002. Guatemala; 2003.<br>• Ministry of Health and Social Assistance (Guatemala), University of Valle (Guatemala) and Division of Reproductive Health, Centers for Disease Control and Prevention (CDC). Guatemala Reproductive Health Survey 2008-2009. Atlanta, United States: Centers for Disease Control and Prevention (CDC); 2008.
Guinea	Yes	Yes	1992	3.3	2012	2.4	4	• Keita, Mohamed Lamine, Mamadou Chérif Bah, Mamadou Badian Diallo, et Bernard Barrére. Enquête Demographique et de la Santé, Guinéé-1992. Conakry, Guinée et Calverton, Mariland US; Direction Nationale de la Statistique et de l'Informatisation; 1994.<br>• Direction Nationale de la Statistique [Guinée] et Macro International Inc. Enquête Démographique et de Santé, Guinée 1999. Calverton, Maryland USA: Direction Nationale de la Statistique et Macro International Inc.; 2000.

Country	Cross sectional analysis (n=150)	Trend analysis (n=121)	First year	First CS (%)	Last year	Last CS (%)	Data points (N)	SOURCES
								• Direction Nationale de la Statistique (DNS) (Guinée) et ORC Macro. Enquête Démographique et de Santé, Guinée 2005. Calverton, Maryland, USA: DNS et ORC Macro; 2006.<br>• Institut National de la Statistique, Ministère du Plan Ministère d'Etat de la Santé et de l'Hygiène Publique Conakry, Guinée Measure DHS. Enquête Démographique et de Santé et à Indicateurs Multiples (EDS-MICS-IV) Guinée 2012. ICF International Calverton, Maryland, U.S.A; 2013.
Guinea-Bissau	Yes	No	-	-	2010	2.3	1	Ministério da Economia, do Plano e Integraçao Regional – Direcçao Geral do Plano. Inquérito aos Indicadores Múltiplos, Inquérito Demográfico de Saúde Reprodutiva 2010. Relatório Final. Guiné-Bissau; 2011.
Guyana	Yes	No	-	-	2009	13.3	1	Ministry of Health (MOH), Bureau of Statistics (BOS), and ICF Macro. Guyana Demographic and Health Survey 2009. Georgetown, Guyana: MOH, BOS, and ICF Macro; 2010.
Haiti	Yes	Yes	1994	1.6	2012	5.5	4	• Cayemittes Michel, Antonio Rival, Bernard Barrère, Gérald Lerebours, Michaèle Amédée Gédéon. Enquête Mortalité, Morbidité et Utilisation des Services (EMMUS-II), Haïti 1994/95. Calverton, Maryland USA: Institut Hattien de l'Enfance et Macro International Inc.; 1995.<br>• Cayemittes Michel, Marie Florence Placide, Bernard Barrère, Soumaïla Mariko, Blaise Sévère. Enquête Mortalité, Morbidité et Utilisation des Services, Haïti 2000. Calverton, Maryland, USA: Ministère de la Santé Publique et de la Population, Institut Haïtien de l'Enfance et ORC Macro; 2001.<br>• Cayemittes Michel, Marie Florence Placide, Soumaïla Mariko, Bernard Barrère, Blaise Sévère, Canez Alexandre. Enquête Mortalité, Morbidité et Utilisation des Services, Haïti, 2005-2006. Calverton, Maryland, USA: Ministère de la Santé Publique et de la Population, Institut Haïtien de l'Enfance et Macro International Inc.; 2007.<br>• Cayemittes M et al. Enquête Mortalité, Morbidité et Utilisation des Services, Haïti, 2012. Calverton, Maryland, USA : MSPP, IHE et ICF International; 2013.

Country	Cross sectional analysis (n=150)	Trend analysis (n=121)	First year	First CS (%)	Last year	Last CS (%)	Data points (N)	SOURCES
Honduras	Yes	Yes	1991	6.4	2011	18.6	5	• Ministerio de Salud Pública. Honduras Encuesta Nacional de Epidemioloía y Salud Familiar (ENESF), 1991/92. Honduras, 1993.<br>• Ministerio de Salud Pública. Honduras Encuesta Nacional de Epidemioloía y Salud Familiar (ENESF), 1996. Informe Final. Honduras, 1997.<br>• Secretaría de Salud de Honduras (SS), la Asociación Hondureña de Planificación de Familia (ASHONPLAFA) y la Agencia de los Estados Unidos para el Desarrollo Internacional (USAID/Honduras). Encuesta Nacional de Epidemiología y Salud Familiar (ENESF-01) y de la Encuesta Nacional de Salud Masculina (ENSM-01) 2001. Informe Final. Honduras, 2002.<br>• Secretaría de Salud [Honduras], Instituto Nacional de Estadística (INE) y Macro International. Encuesta Nacional de Salud y Demografía 2005-2006. Tegucigalpa, Honduras: SS, INE y Macro International; 2006.<br>• Secretaría de Salud [Honduras], Instituto Nacional de Estadística (INE) e ICF International. Encuesta Nacional de Salud y Demografía 2011-2012. Tegucigalpa, Honduras: SS, INE e ICF International; 2013.
Hungary	Yes	Yes	1994	12.6	2011	33.4	18	European Health for All Database (HFA-DB) [online database updated on April 2014]. WHO Regional Office for Europe; 2014 (http://data.euro.who.int/hfadb/, accessed 15 April 2015).
Iceland	Yes	Yes	1990	11.8	2009	16.2	20	European Health for All Database (HFA-DB) [online database updated on April 2014]. WHO Regional Office for Europe; 2014 (http://data.euro.who.int/hfadb/, accessed 15 April 2015).
India	Yes	Yes	1992	2.5	2008	8.2	4	• International Institute for Population Sciences (IIPS). National Family Health Survey (MCH and Family Planning), India 1992-93. Bombay, India: IIPS; 1995.<br>• International Institute for Population Sciences (IIPS) and ORC Macro. National Family Health Survey (NFHS-2), 1998–99: India. Mumbai, India: IIPS; 2000.<br>• International Institute for Population Sciences (IIPS) and Macro International. National Family Health Survey (NFHS-3), 2005–06: India: Volume I. Mumbai: IIPS; 2007.<br>• International Institute for Population Sciences (IIPS), 2010. District level Household and Facility Survey (DLHS-3), 2007-08: India. Mumbai: IIPS; 2010.

Country	Cross sectional analysis (n=150)	Trend analysis (n=121)	First year	First CS (%)	Last year	Last CS (%)	Data points (N)	SOURCES
Indonesia	Yes	Yes	1991	1.3	2012	12.3	6	• Central Bureau of Statistics, National Family Planning Coordinating Board, Ministry of Health, Macro International Inc. Indonesia Demographic and Health Survey 1991. Columbia, Maryland, USA: Macro International Inc.; 1992.<br>• Central Bureau of Statistics (CBS) [Indonesia] and State Ministry of Population/National Family Planning Coordinating Board (NFPCB) and Ministry of Health (MOH) and Macro International Inc. (MI). Indonesia Demographic and Health Survey 1994. Calverton, Maryland: CBS and MI; 1995.<br>• Central Bureau of Statistics (CBS) [Indonesia] and State Ministry of Population/National Family Planning Coordinating Board (NFPCB) and Ministry of Health (MOH) and Macro International Inc. (MI). Indonesia Demographic and Health Survey 1997. Calverton, Maryland: CBS and MI; 1998.<br>• Badan Pusat Statistik-Statistics Indonesia (BPS) and ORC Macro. Indonesia Demographic and Health Survey 2002-2003. Calverton, Maryland, USA: BPS and ORC Macro; 2003.<br>• Statistics Indonesia (Badan Pusat Statistik-BPS) and Macro International. Indonesia Demographic and Health Survey 2007. Calverton, Maryland, USA: BPS and Macro International; 2008.<br>• Statistics Indonesia (Badan Pusat Statistik—BPS), National Population and Family Planning Board (BKKBN), and Kementerian Kesehatan (Kemenkes—MOH), and ICF International. Indonesia Demographic and Health Survey 2012. Jakarta, Indonesia: BPS, BKKBN, Kemenkes, and ICF International; 2013.
Iran (Islamic Republic of)	Yes	Yes	2000	35	2009	47.9	4	Bahadori F, Hakimi S, Heidarzade M. The trend of caesarean delivery in the Islamic Republic of Iran. EMHJ. 2013;19(S3):S67-S70.
Iraq	Yes	No	-	-	2011	22.2	1	The Central Statistics Organization and the Kurdistan Regional Statistics Office. Iraq Multiple Indicator Cluster Survey 2011. Final Report. Baghdad, Iraq: The Central Statistics Organization and the Kurdistan Regional Statistics Office; 2012.

Country	Cross sectional analysis (n=150)	Trend analysis (n=121)	First year	First CS (%)	Last year	Last CS (%)	Data points (N)	SOURCES
Ireland	Yes	Yes	1990	10.5	2010	26.6	20	European Health for All Database (HFA-DB) [online database updated on April 2014]. WHO Regional Office for Europe; 2014 (http://data.euro.who.int/hfadb/, accessed 15 April 2015).
Israel	Yes	Yes	1989	9.9	2011	19.8	20	European Health for All Database (HFA-DB) [online database updated on April 2014]. WHO Regional Office for Europe; 2014 (http://data.euro.who.int/hfadb/, accessed 15 April 2015).
Italy	Yes	Yes	1990	20.8	2011	38.1	22	European Health for All Database (HFA-DB) [online database updated on April 2014]. WHO Regional Office for Europe; 2014 (http://data.euro.who.int/hfadb/, accessed 15 April 2015).
Jamaica	Yes	No[#]	2002	9.6	2011	21.2	3	Statistical Institute of Jamaica (STATIN) and UNICEF. Jamaica Multiple Indicator Cluster Survey 2011: Final Report. Kingston, Jamaica: STATIN and UNICEF; 2013.
Japan	Yes	Yes	1990	10.0	2011	19.2	8	(Ministry of Health, Labour and Welfare. Summary of Health Care Facilities and Hospital Statistics 2011 (平成23 年（2011）医療施設(静態?動態)調査?病院報告の概況).
Jordan	Yes	Yes	1990	5.7	2012	28	5	• Department of Statistics, Ministry of Health and IRD/Macro International Inc. Jordan Population and Family Health Survey 1990. Columbia, Maryland USA: IRD/Macro International Inc.; 1992.<br>• Department of Statistics (DOS) [Jordan] and Macro International Inc. (MI). Jordan Population and Family Health Survey 1997. Calverton, Maryland: DOS and MI; 1998.<br>• Department of Statistics [Jordan] and ORC Macro. Jordan Population and Family Health Survey 2002. Calverton, Maryland, USA: Department of Statistics and ORC Macro; 2003.<br>• Department of Statistics [Jordan] and Macro International Inc. Jordan Population and Family Health Survey 2007. Calverton, Maryland, USA: Department of Statistics and Macro International Inc.; 2008.<br>• Department of Statistics [Jordan] and ICF International. Jordan Population and Family Health Survey 2012. Calverton, Maryland, USA: Department of Statistics and ICF International; 2013.
Kazakhstan	Yes	Yes	1990	4.6	2012	15.2	23	European Health for All Database (HFA-DB) [online database updated on April 2014]. WHO Regional Office for Europe; 2014 (http://data.euro.who.int/hfadb/

Country	Cross sectional analysis (n=150)	Trend analysis (n=121)	First year	First CS (%)	Last year	Last CS (%)	Data points (N)	SOURCES
								, accessed 15 April 2015).
Kenya	Yes	Yes	1993	5.2	2008	6.2	4	• National Council for Population and Development (NCPD), Central Bureau of Statistics (CBS) (Office of the Vice President and Ministry of Planning and National Development [Kenya]), and Macro International Inc. (MI). Kenya Demographic and Health Survey 1993. Calverton, Maryland: NCPD, CBS, and MI; 1994.<br>• National Council for Population and Development (NCPD), Central Bureau of Statistics (CBS) (Office of the Vice President and Ministry of Planning and National Development) [Kenya], and Macro International Inc. (MI). Kenya Demographic and Health Survey 1998. Calverton, Maryland: NDPD, CBS, and MI; 1999.<br>• Central Bureau of Statistics (CBS) [Kenya], Ministry of Health (MOH) [Kenya], and ORC Macro. Kenya Demographic and Health Survey 2003. Calverton, Maryland: CBS, MOH, and ORC Macro; 2004.<br>• Kenya National Bureau of Statistics (KNBS) and ICF Macro. Kenya Demographic and Health Survey 2008-09. Calverton, Maryland: KNBS and ICF Macro; 2010.
Kyrgyzstan	Yes	Yes	1990	3.1	2014	7.4	23	• National Statistical Committee of the Kyrgyz Republic and UNICEF. Kyrgyzstan Multiple Indicator Cluster Survey 2014, Key Findings. Bishkek, Kyrgyzstan: National Statistical Committee of the Kyrgyz Republic and UNICEF; 2014.<br>• European Health for All Database (HFA-DB) [online database updated on April 2014]. WHO Regional Office for Europe; 2014 (http://data.euro.who.int/hfadb/, accessed 28 June 2014).
Lao People's Dem. Republic	Yes	No	-	-	2012	3.7	1	Ministry of Health (MoH), Lao Statistics Bureau (LSB). Lao Social Indicator Survey LSIS (MICS/DHS) 2011-12 (Multiple Indicator Cluster Survey/Demographic and Health Survey). Ministry of Health and Lao Statistics Bureau; 2012.
Latvia	Yes	Yes	1990	7.0	2011	23	22	European Health for All Database (HFA-DB) [online database updated on April 2014]. WHO Regional Office for Europe; 2014 (http://data.euro.who.int/hfadb/, accessed 15 April 2015).

Country	Cross sectional analysis (n=150)	Trend analysis (n=121)	First year	First CS (%)	Last year	Last CS (%)	Data points (N)	SOURCES
Lebanon	No*	No#	1995	15.1	2004	23.2	-	Lebanon Health Survey 2004 Report. The Pan Arab Project for Family Health. http://www.pdslebanon.org/UserFiles/File/Lebanon%20Report%20English(1).pdf
Lesotho	Yes	No	-	-	2009	6.7	1	Ministry of Health and Social Welfare (MOHSW) [Lesotho] and ICF Macro. Lesotho Demographic and Health Survey 2009. Maseru, Lesotho: MOHSW and ICF Macro; 2010.
Liberia	Yes	No	-	-	2007	3.5	1	Liberia Institute of Statistics and Geo-Information Services (LISGIS) [Liberia], Ministry of Health and Social Welfare [Liberia], National AIDS Control Program [Liberia], and Macro International Inc. Liberia Demographic and Health Survey 2007. Monrovia, Liberia: Liberia Institute of Statistics and Geo-Information Services (LISGIS) and Macro International Inc.; 2008.
Lithuania	Yes	Yes	1992	8.3	2011	23.2	20	European Health for All Database (HFA-DB) [online database updated on April 2014]. WHO Regional Office for Europe; 2014 (http://data.euro.who.int/hfadb/, accessed 15 April 2015).
Luxembourg	Yes	Yes	1996	15.9	2011	27.4	16	European Health for All Database (HFA-DB) [online database updated on April 2014]. WHO Regional Office for Europe; 2014 (http://data.euro.who.int/hfadb/, accessed 15 April 2015).
Madagascar	Yes	Yes	1992	1	2013	1.9	5	• Centre National de Recherches sur l'Environnement, Macro International Inc. Enquête Nationale Dêmographique et Sanitaire Madagascar en 1992. Calverton, Maryland USA: Macro International Inc.; 1994.<br>• Direction de la Démographie et des Statistiques Sociales, Institut National de la Statistique (INSTAT) [Madagascar] et Macro International Inc. Enquête Démographique et de Santé, Madagascar 1997. Calverton, Maryland, USA: INSTAT et Macro International Inc.; 1998.<br>• Institut National de la Statistique (INSTAT) et ORC Macro. Enquête Démographique et de Santé de Madagascar 2003-2004. Calverton, Maryland, USA: INSTAT et ORC Macro; 2005.<br>• Institut National de la Statistique (INSTAT) et ICF Macro. Enquête Démographique et de Santé de Madagascar 2008-2009. Antananarivo, Madagascar: INSTAT et ICF Macro; 2010.<br>• l'Institut National de la Statistique, l'Office National de Nutrition (ONN). L'Enquête Nationale sur le Suivi des indicateurs des Objectifs du Millénaire

Country	Cross sectional analysis (n=150)	Trend analysis (n=121)	First year	First CS (%)	Last year	Last CS (%)	Data points (N)	SOURCES
								pour le Développement 2012-2013 (ENSOMD). Madagascar; 2014.
Malawi	Yes	Yes	1992	3.4	2014	5.1	5	• National Statistical Office and Macro International Inc. Malawi Demographic and Health Survey 1992. Calverton, Maryland USA: Macro International Inc.; 1994.<br>• National Statistical Office [Malawi] and ORC Macro. Malawi Demographic and Health Survey 2000. Zomba, Malawi and Calverton, Maryland, USA: National Statistical Office and ORC Macro; 2001.<br>• National Statistical Office (NSO) [Malawi], and ORC Macro. Malawi Demographic and Health Survey 2004. Calverton, Maryland, USA: NSO and ORC Macro; 2005.<br>• National Statistical Office (NSO), and ICF Macro. Malawi Demographic and Health Survey 2010. Zomba, Malawi, and Calverton, Maryland, USA: NSO and ICF Macro; 2011.<br>• National Statistical Office. Malawi MDG Endline Survey 2014, Key Findings. Zomba, Malawi: National Statistical Office; 2014
Malaysia	Yes	No	-	-	2006	15.7	1	Ravindran J. Rising caesarean section rates in public hospitals in Malaysia 2006. Med J Malaysia. 2008;63(5):434-5.
Maldives	Yes	No	-	-	2011	41.1	1	Ministry of Health. The Maldives Health Statistics 2012. Male, Republic of Maldives;2013.
Mali	Yes	Yes	1995	0.8	2013	2.7	4	• Coulibaly, Salif, Fatoumata Dicko, Seydou Moussa Traoré, Ousmane Sidibé, Michka Seroussi et Bernard Barrère. Enquête Démographique et de Santé, Mali 1995-1996. Calverton, Maryland, USA: Cellule de Planification et de Statistique du Ministère de la Santé), Direction Nationale de la Statistique et de l'Informatique et Macro International Inc.; 1996.<br>• Cellule de Planification et de Statistique du Ministère de la Santé (CPS/MS), Direction Nationale de la Statistique et de l'Informatique (DNSI) et ORC Macro. Enquête Démographique et de Santé au Mali 2001. Calverton, Maryland, USA: CPS/MS, DNSI et ORC Macro; 2002.<br>• Cellule de Planification et de Statistique du Ministère de la Santé (CPS/MS), Direction Nationale de la Statistique et de l'Informatique du Ministère de

Country	Cross sectional analysis (n=150)	Trend analysis (n=121)	First year	First CS (%)	Last year	Last CS (%)	Data points (N)	SOURCES
								l'Économie, de l'Industrie et du Commerce (DNSI/MEIC) et Macro International Inc. Enquête Démographique et de Santé du Mali 2006. Calverton, Maryland, USA: CPS/DNSI et Macro International Inc.; 2007.<br>• Cellule de Planification et de Statistique (CPS/SSDSPF), Institut National de la Statistique (INSTAT/MPATP), INFO-STAT et ICF International. Enquête Démographique et de Santé au Mali 2012-2013. Rockville, Maryland, USA : CPS, INSTAT, INFO-STAT et ICF International; 2014.
Malta	Yes	Yes	1995	18.2	2011	33.5	17	European Health for All Database (HFA-DB) [online database updated on April 2014]. WHO Regional Office for Europe; 2014 (http://data.euro.who.int/hfadb/ , accessed 15 April 2015).
Mauritania	Yes	Yes	2001	3.3	2011	9.6	1	• Office National de la Statistique (ONS) [Mauritanie] et ORC Macro. Enquête Démographique et de Santé Mauritanie 2000-2001. Calverton, Maryland, USA : ONS et ORC Macro; 2001.<br>• Office National de la Statistique, UNICEF. Mauritanie Enquête par grappes à indicateurs multiples 2011. Rapport Final. Mauritanie; 2014.
Mexico	Yes	Yes	1987	12.4	2012	45.2	5	• Dirección General de Planificación Familiar, Institute for Resource Development/Macro Systems. Mexico Encuesta Nacional sobre Fecundidad y Salud 1987. Dirección General de Planificación Familiar, Institute for Resource Development/Macro Systems, Columbia, Maryland USA, 1989.<br>• Instituto Nacional de Salud Pública. Encuesta Nacional de Salud 2000. Instituto Nacional de Salud Pública. Cuernavaca, Morelos, México, 2003.<br>• Chavez Galindo AM, Uribe Zúñiga P, Palma Cabrera Y. La Salud Reproductiva en México. Análisis de la Encuesta Nacional de Salud Reproductiva 2003. Secretaría de Salud, Centro Regional de Investigaciones Multidisciplicarias, UNAM. México, 2007.<br>• Instituto Nacional de Estadística y Geografía. Mujeres y hombres en México 2009. Instituto Nacional de Estadística y Geografía. Aguascalientes, 2009.<br>• Instituto Nacional de Estadística y Geografía. Encuesta Nacional de la Dinámica Demográfica 2009. Panorama sociodemográfico de México. Principales Resultados. Instituto Nacional de Estadística y Geografía. Aguascalientes, 2011.<br>• Encuesta Nacional de Salud y Nutrición 2012. Resultados Nacionales.

Country	Cross sectional analysis (n=150)	Trend analysis (n=121)	First year	First CS (%)	Last year	Last CS (%)	Data points (N)	SOURCES
								Instituto Nacional de Salud PublicaMexico. Mexico, 2013.
Mongolia	Yes	Yes	1998	5.1	2013	23.4	5	• National Statistics Office, United Nations Population Fund. Mongolia National Report Reproductive Health Survey 1998. National Statistics Office, United Nations Population Fund, Ministry of Health. Ulaanbaatar, Mongolia, 1999.<br>• National Statistics Office, United Nations Population Fund. Mongolia National Report Reproductive Health Survey 2003. National Statistics Office, United Nations Population Fund, Ministry of Health. Ulaanbaatar, Mongolia, 2004.<br>• National Statistics Office, United Nations Population Fund, Ministry of Health. Mongolia Reproductive Health Survey 2008. National Report. National Statistics Office, United Nations Population Fund, Ministry of Health. Ulaanbaatar, Mongolia, 2009.<br>• National Statistics Office. Mongolia Child Development -2010 Survey. Multiple Indicator Cluster Survey -4. Final Report. National Statistics Office. Ulaanbaatar, Mongolia, 2013.<br>• National Statistics Office of Mongolia, UNFPA, UNICEF. Mongolia Social Indicator Sample Survey (SISS) 2013. Key Findings, Ulaanbaatar, Mongolia; 2014.
Montenegro	Yes	Yes	1997	9.0	2010	23.6	8	European Health for All Database (HFA-DB) [online database updated on April 2014]. WHO Regional Office for Europe; 2014 (http://data.euro.who.int/hfadb/ , accessed 15 April 2015).
Morocco	Yes	Yes	1992	2.0	2011	16.0	4	• Ministère de la Santé Publique, Macro International Inc. Enquête Nationale sur la Population et la Santé (ENPS-II) 1992. Columbia, Maryland, USA: Macro International Inc.; 1993.<br>• Azelmat, Mustapha, Mohamed Ayad et El Arbi Housni. Enquête de Panel sur la Population et la Santé (EPPS) 1995. Calverton, Maryland, USA: Ministère de la Santé Publique, Direction de la Planification et des Ressources Financières, Service des Etudes et de l'Information Sanitaire et Macro International Inc.; 1996.<br>• Ministère de la Santé [Maroc], ORC Macro, et Ligue des États Arabes. Enquête sur la Population et la Santé Familiale (EPSF) 2003-2004. Calverton,

Country	Cross sectional analysis (n=150)	Trend analysis (n=121)	First year	First CS (%)	Last year	Last CS (%)	Data points (N)	SOURCES
								Maryland, USA: Ministère de la Santé et ORC Macro; 2005.<br>• Ministère de la Santé, PAPFAM. Enquête Nationale sur la Population et la Santé Familiale (ENPSF) 2011. Maroc; 2012.
Mozambique	Yes	Yes	1997	2.7	2011	3.9	3	• Moçambique Inquérito Demográfico e de Saúde 2003. Instituto Nacional de Estatística, Macro International Inc. Calverton, Maryland, USA: Macro International Inc., 1998.<br>• Moçambique Inquérito Demográfico e de Saúde 2003. Instituto Nacional de Estatística, Ministério da Saúde. ORC Macro. Calverton MD, USA: ORC Macro. 2005. Ministério da Saúde. ORC Macro. Calverton MD, USA: ORC Macro. 2005.<br>• Ministerio da Saude (MISAU), Instituto Nacional de Estatística (INE) e ICF International (ICFI). Moçambique Inquérito Demográfico e de Saúde 2011. Calverton, Maryland, USA: MISAU, INE e ICFI; 2013.
Namibia	Yes	Yes	1992	6.9	2013	14.4	3	• Ministry of Health and Social Services (MoHSS) [Namibia] and Macro International Inc. Namibia Demographic and Health Survey 1992. Calverton, Maryland USA: MoHSS and Macro International Inc.; 1993.<br>• Ministry of Health and Social Services (MoHSS) [Namibia] and Macro International Inc. Namibia Demographic and Health Survey 2006-07. Windhoek, Namibia and Calverton, Maryland, USA: MoHSS and Macro International Inc.; 2008.<br>• The Namibia Ministry of Health and Social Services (MoHSS) and ICF International. The Namibia Demographic and Health Survey 2013. Windhoek, Namibia, and Rockville, Maryland, USA: MoHSS and ICF International; 2014.
Nepal	Yes	Yes	1996	1	2011	4.6	4	• Family Health Division, Ministry of Health, New ERA, Macro International Inc. Nepal Family Health Survey 1996. Calverton, Maryland, USA: Macro International Inc., 1997.<br>• Ministry of Health [Nepal], New ERA, and ORC Macro. Nepal Demographic and Health Survey 2001. Calverton, Maryland, USA: Family Health Division, Ministry of Health; New ERA; and ORC Macro, 2002.<br>• Ministry of Health and Population (MOHP) [Nepal], New ERA, and Macro International Inc. Nepal Demographic and Health Survey 2006. Kathmandu,

Country	Cross sectional analysis (n=150)	Trend analysis (n=121)	First year	First CS (%)	Last year	Last CS (%)	Data points (N)	SOURCES
								Nepal: Ministry of Health and Population, New ERA, and Macro International Inc., 2007.<br>• Ministry of Health and Population (MOHP) [Nepal], New ERA, and ICF International Inc. Nepal Demographic and Health Survey 2011. Kathmandu, Nepal: Ministry of Health and Population, New ERA, and ICF International, Calverton, Maryland; 2012.
Netherlands	Yes	Yes	1990	7.5	2010	15.6	19	European Health for All Database (HFA-DB) [online database updated on April 2014]. WHO Regional Office for Europe; 2014 (http://data.euro.who.int/hfadb/ , accessed 15 April 2015).
New Zealand	Yes	Yes	1996	20.8	2012	33.4	16	National Women's Annual Clinical Report 2012 at http://www.adhb.govt.nz/nwhealthinfo p 86
Nicaragua	Yes	Yes	1993	13.5	2013	29.7	1	• Asociacion Pro-Bienestar de la Familia Nicaragüense - PROFAMILIA, CDC. Encuesta sobre Salud Familiar Nicaragua 92-93. Managua, Nicaragua, 1993.<br>• Instituto Nacional de Estadísticas y Censos (INEC), Ministerio de Salud (MINSA), Macro Internatioanl Inc. Encuesta Nicaragüense de Demografía y Salud 1998. Calverton, Maryland, USA: Macro Internatioanl Inc., 1999.<br>• Instituto Nacional de Estadísticas y Censos (INEC), Ministerio de Salud (MINSA), ORC Macro Encuesta Nicaragüense de Demografía y Salud 2001. Calverton, USA: ORC Macro, 2002.<br>• Instituto Nacional de Información de Desarrollo (INIDE), Ministerio de Salud (MINSA). Encuesta Nicaragüense de Demografía y Salud ENDESA 2006/07. Informe Final. Nicaragua, 2008.<br>• Instituto Nacional de Información de Desarrollo (INIDE) Ministerio de Salud (MINSA). Encuesta Nicaragüense de Demografía y Salud 2011/12. Informe Preliminar. Ministerio de Salud, Nicaragua; 2013.
Niger	Yes	Yes	1992	0.9	2012	1.4	4	• Direction de la Statistique et des Comptes Natinaux, Direction Général du Plan, Ministère des Finances et du Plan, Macro International Inc. Enquête Démographique et de Santé Niger 1992. Columbia, Maryland, USA: Macro International Inc.; 1993.<br>• Attama, Sabine, Michka Seroussi, Alichina Idrissa Kourguéni, Harouna Koché et Bernard Barrèm. Enquête Démographique et de Santé, Niger 1998. Calverton, Maryland, USA: Care International Niger et Macro International

Country	Cross sectional analysis (n=150)	Trend analysis (n=121)	First year	First CS (%)	Last year	Last CS (%)	Data points (N)	SOURCES
								Inc.; 1999.<br>• Institut National de la Statistique (INS) et Macro International Inc. Enquête Démographique et de Santé et à Indicateurs Multiples du Niger 2006. Calverton, Maryland, USA: INS et Macro International Inc.; 2007.<br>• Institut National de la Statistique (INS) et ICF International. Enquête Démographique et de Santé et à Indicateurs Multiples du Niger 2012. Calverton, Maryland, USA : INS et ICF International; 2013.
Nigeria	Yes	Yes	1990	2.9	2013	2.0	5	• Federal Office of Statistics, IRD/Macro International Inc. Nigeria Demographic and Health Survey 1990. Colombia, Maryland, USA: IRD/Macro International Inc.; 1992.<br>• National Population Commission [Nigeria]. Nigeria Demographic and Health Survey 1999. Calverton, Maryland: National Population Commission and ORC/Macro; 2000.<br>• National Population Commission (NPC) [Nigeria] and ORC Macro. Nigeria Demographic and Health Survey 2003. Calverton, Maryland: National Population Commission and ORC Macro; 2004.<br>• National Population Commission (NPC) [Nigeria] and IF Macro. Nigeria Demographic and Health Survey 2008. Abuja, Nigeria: National Population Commission and ICF Macro; 2009.<br>• National Population Commission (NPC) [Nigeria] and ICF International. Nigeria Demographic and Health Survey 2013. Abuja, Nigeria, and Rockville, Maryland, USA: NPC and ICF International; 2014.
Norway	Yes	Yes	1990	12.7	2009	17.3	20	European Health for All Database (HFA-DB) [online database updated on April 2014]. WHO Regional Office for Europe; 2014 (http://data.euro.who.int/hfadb/, accessed 15 April 2015).
Oman	Yes	Yes	1995	6.6	2011	17.0	10	• Jurdi R, Khawaja M. Caesarean section rates in the Arab region: a cross-national study. Health Policy Plan. 2004;19(2):101-10.<br>• National Health Survey 2000, Ministry of Health Sultanate of Oman, UNICEF and UNFPA.<br>• Ministry of Health. Department of Health Information and Statistics, Directorate General of Planning. Annual Health Report 2003. Oman; 2003.<br>• Ministry of Health. Department of Health Information and Statistics,

Country	Cross sectional analysis (n=150)	Trend analysis (n=121)	First year	First CS (%)	Last year	Last CS (%)	Data points (N)	SOURCES
								Directorate General of Planning. Annual Health Report 2004. Oman; 2004.<br>• Ministry of Health. Department of Health Information and Statistics, Directorate General of Planning. Annual Health Report 2005. Oman; 2005.<br>• Ministry of Health. Department of Health Information and Statistics, Directorate General of Planning. Annual Health Report 2006. Oman; 2006.<br>• Ministry of Health. Department of Health Information and Statistics, Directorate General of Planning. Annual Health Report 2007. Oman; 2007.<br>• Ministry of Health. Department of Health Information and Statistics, Directorate General of Planning. Annual Health Report 2008. Oman; 2008.<br>• Ministry of Health. Department of Health Information and Statistics, Directorate General of Planning. Annual Health Report 2009. Oman; 2009.<br>• Ministry of Health. Department of Health Information and Statistics, Directorate General of Planning. Annual Health Report 2010. Oman; 2010.<br>• Ministry of Health. Department of Health Information and Statistics, Directorate General of Planning. Annual Health Report 2011. Oman; 2012.
Pakistan	Yes	Yes	1991	2.7	2012	14.1	3	• National Institute of Population Studies, IRD/Macro International Inc. Pakistan Demographic Health survey 1990/1991. Columbia, Maryland, USA: IRD/Macro International Inc.; 1992.<br>• National Institute of Population Studies (NIPS) [Pakistan], and Macro International Inc. Pakistan Demographic and Health Survey 2006-07. Islamabad, Pakistan: National Institute of Population Studies and Macro International Inc.; 2008.<br>• National Institute of Population Studies (NIPS) [Pakistan] and ICF International. Pakistan Demographic and Health Survey 2012-13. Islamabad, Pakistan, and Calverton, Maryland, USA: NIPS and ICF International; 2013.
Panama	Yes	No	-	-	2013	27.7	1	• Ruth Graciela De León Richardson et al.: Instituto Commemorative Gorgas de Estudios de la Salud. Panamá Encuesta Nacional de Salud Sexual y Reproductiva 2009 (ENASSER 2009)- Informe Final. Panamá; 2011.<br>• Contraloría General de la República. Encuesta de Indicadores Múltiples por Conglomerados de Panamá 2013, Resultados Principales Panamá, Panamá: Contraloría General; 2014.

Country	Cross sectional analysis (n=150)	Trend analysis (n=121)	First year	First CS (%)	Last year	Last CS (%)	Data points (N)	SOURCES
Paraguay	Yes	Yes	1990	13	2008	33.1	4	• Centro Paraguayo de Estudios de Población, Macro Systems Inc. Encuesta Nacional de Demografía y Salud 1990. Columbia, maryland, USA: Macro Systems Inc.; 1991.<br>• Centro Paraguay de Estudios de Poblacíon (CEPEP), Centers fro Diease Control and Prevention (CDC), United States Agency for International Development (USAID). Encuesta Nacional de Salud Materno Infantil: 1998 (ENSMI-98). Informe Final. Asunción, Paraguay; 1999.<br>• Centro Paraguay de Estudios de Poblacíon (CEPEP), USAID, UNPFA, CDC, IPPF. Encuesta Nacional de Demografía y Salud Reproductiva 2004 (ENDSSR 2004). Informe Final. Asunción, Paraguay; 2005.<br>• Centro Paraguay de Estudios de Poblacíon (CEPEP), USAID, UNPFA, CDC, IPPF. Encuesta Nacional de Demografía y Salud Reproductiva 2008 (ENDSSR 2008). Informe Final. Asunción, Paraguay; 2009.
Peru	Yes	Yes	1991	10	2013	26.5	9	• Nacional de Estadística e Informática (INEI), Asociación Benéfica PRISMA, Macro International Inc. Encuesta Demográfica y de Salud Familiar (ENDES 1991-1992). Columbia, Maryland, USA: Macro International Inc.; 1992.<br>• Instituto Nacional de Estadística e Informática, Macro International Inc. Peru Encuesta Demográfica y de Salud Familiar 1996. Calverton, Maryland, USA: Macro International Inc.; 1997.<br>• Instituto Nacional de Estadística e Informática, Macro International Inc. Peru Encuesta Demográfica y de Salud Familiar 2000. Lima, Peru; 2001.<br>• Instituto Nacional de Estadística e Informática, ORC Macro. Peru Encuesta Demográfica y de Salud Familiar - ENDES Continua, 2004-2006. Calverton, MD, USA: ORC Macro; 2007.<br>• Instituto Nacional de Estadística e Informática, ORC Macro. Peru Encuesta Demográfica y de Salud Familiar 2007-2008 -Informe Pribcipal. Calverton, MD, USA: ORC Macro; 2009.<br>• Instituto Nacional de Estadística e Informática, ORC Macro. Peru Encuesta Demográfica y de Salud Familiar - ENDES Continua, 2009 Calverton, MD, USA: ORC Macro; 2010.<br>• Instituto Nacional de Estadística e Informática, ORC Macro. Peru Encuesta Demográfica y de Salud Familiar - ENDES Continua, 2010 Calverton, MD,

Country	Cross sectional analysis (n=150)	Trend analysis (n=121)	First year	First CS (%)	Last year	Last CS (%)	Data points (N)	SOURCES
								USA: ORC Macro; 2011.<br>• Instituto Nacional de Estadística e Informática, ORC Macro. Peru Encuesta Demográfica y de Salud Familiar, 2011 Calverton, MD, USA: ORC Macro; 2012.<br>• Instituto Nacional de Estadística e Informática, ORC Macro. Peru Encuesta Demográfica y de Salud Familiar 2013. Calverton, MD, USA: ORC Macro; 2014.
Philippines	Yes	Yes	1993	5.9	2014	9.3	6	• National Statistics Office (NSO) [Philippines] and Macro International Inc. (MI). National Demographic Survey 1993. Calverton, Maryland: NSO and MI; 1994.<br>• National Statistics Office (NSO)], Department of Health (DOH) [Philippines] and Macro International Inc. (MI). National Demographic and Health Survey 1998. Manila: NSO and MI; 1999.<br>• National Statistics Office (NSO) [Philippines], and ORC Macro. National Demographic and Health Survey 2003. Calverton, Maryland: NSO and ORC Macro; 2004.<br>• National Statistics Office (NSO) [Philippines], and ICF Macro. National Demographic and Health Survey 2008. Calverton, Maryland: National Statistics Office and ICF Macro; 2009.<br>• The National Statistics Office (NSO). 2011 Family Health Survey (FHS). NSO: Manila, Philippines; 2012.<br>• Philippine Statistics Authority (PSA) [Philippines], and ICF International. Philippines National Demographic and Health Survey 2013. Manila, Philippines, and Rockville, Maryland, USA: PSA and ICF International; 2014.
Poland	Yes	Yes	1994	13.8	2011	29.9	8	European Health for All Database (HFA-DB) [online database updated on April 2014]. WHO Regional Office for Europe; 2014 (http://data.euro.who.int/hfadb/, accessed 15 April 2015).
Portugal	Yes	Yes	1990	18.6	2010	36.2	20	WHO/European Regional Office Health for all Database (Internet communication of 4 October 2012 at http://data.euro.who.int/hfadb)
Qatar	Yes	Yes	1998	15.9	2012	19.5	2	• Jurdi R, Khawaja M. Caesarean section rates in the Arab region: a cross-national study. Health Policy Plan. 2004;19(2): 101-10.<br>• Ministry of Development Planning and Statistics, Qatar Foundation,

Country	Cross sectional analysis (n=150)	Trend analysis (n=121)	First year	First CS (%)	Last year	Last CS (%)	Data points (N)	SOURCES
								Supreme Council of Health and UNICEF. Multiple Indicator Cluster Survey in the State of Qatar 2012. Doha, Qatar; 2014.
Republic of Korea	Yes	Yes	1991	17.3	2009	36.6	7	• Korea Institute for Health and Social Affairs. The 2003 National Survey on Fertility, Family Health and Welfare in Korea. Republic of Korea; 2004.<br>• Korea Institute for Health and Social Affairs. The 2009 National Survey on Fertility, Family Health and Welfare in Korea. Republic of Korea; 2009.<br>• Korea Institute for Health and Social Affairs. The 2012 National Survey on Fertility, Family Health and Welfare in Korea. Republic of Korea; 2012.
Republic of Moldova	Yes	Yes	1990	5.3	2012	15.8	23	European Health for All Database (HFA-DB) [online database updated on April 2014]. WHO Regional Office for Europe; 2014 (http://data.euro.who.int/hfadb/, accessed 15 April 2015).
Romania	Yes	Yes	1992	7.2	2011	36.3	20	European Health for All Database (HFA-DB) [online database updated on April 2014]. WHO Regional Office for Europe; 2014 (http://data.euro.who.int/hfadb/, accessed 15 April 2015).
Russian Federation	Yes	Yes	1990	6.7	2010	22.1	18	European Health for All Database (HFA-DB) [online database updated on April 2014]. WHO Regional Office for Europe; 2014 (http://data.euro.who.int/hfadb/, accessed 15 April 2015).
Rwanda	Yes	Yes	1992	1.8	2010	7.1	4	• Office National de la Population, Macro International Inc. Enquête Démographic et de Santé 1992. Calverton, Maryland, USA: Macro International Inc.; 1994.<br>• Office National de la Population (ONAPO) [Rwanda] et ORC Macro. Enquête Démographique et de Santé, Rwanda 2000. Kigali, Rwanda et Calverton, Maryland, USA: Ministère de la Santé, Office National de la Population et ORC Macro; 2001.<br>• Institut National de la Statistique du Rwanda (INSR) and ORC Macro. Rwanda Demographic and Health Survey 2005. Calverton, Maryland, USA: INSR and ORC Macro; 2006.<br>• National Institute of Statistics of Rwanda (NISR) [Rwanda], Ministry of Health (MOH) [Rwanda], and ICF International. Rwanda Demographic and Health Survey 2010. Calverton, Maryland, USA: NISR, MOH, and ICF International; 2012.

Country	Cross sectional analysis (n=150)	Trend analysis (n=121)	First year	First CS (%)	Last year	Last CS (%)	Data points (N)	SOURCES
Samoa	Yes	No	-	-	2009	12.8	1	Ministry of Health [Samoa], Bureau of Statistics [Samoa], and ICF Macro. Samoa Demographic and Health Survey 2009. Apia, Samoa: Ministry of Health, Samoa; 2010.
Saudi Arabia	Yes	Yes	1996	8.1	2012	22.3	8	• Ministry of Health, General Directorate of Statistics. Health Statistics Book for the year of 2006. (1427) Saudia Arabia, 2006.<br>• Ministry of Health, General Directorate of Statistics. Health Statistics Book for the year of 2006. (1428) Saudia Arabia, 2007.<br>• Ministry of Health, General Directorate of Statistics. Health Statistics Book for the year of 2006. (1429) Saudia Arabia, 2008.<br>• Ministry of Health, General Directorate of Statistics. Health Statistics Book for the year of 2006. (1430) Saudia Arabia, 2009.<br>• Ministry of Health, General Directorate of Statistics. Health Statistics Book for the year of 2006. (1431) Saudia Arabia, 2010.<br>• Ministry of Health, General Directorate of Statistics. Health Statistics Book for the year of 2012 (1433). Saudi Arabia; 2013.
Senegal	Yes	Yes	1992	2.3	2012	3.8	4	• Ministère de l'Economie des Finances et du Plan, Macro International Inc. Enquête Démographic et de Santé au Sénégal (EDS-II) 1992/93. Calverton, Maryland, USA: Macro International Inc.; 1994.<br>• Ndiaye, Salif, et Mohamed Ayad. Enquête Démographique et de Santé au Sénégal 2005. Calverton, Maryland, USA: Centre de Recherche pour le Développement Humain [Sénégal] et ORC Macro; 2006.<br>• Agence Nationale de la Statistique et de la Démographie (ANSD) [Sénégal], et ICF International. Enquête Démographique et de Santé à Indicateurs Multiples au Sénégal (EDS-MICS) 2010-2011. Calverton, Maryland, USA: ANSD et ICF International; 2012.<br>• Agence Nationale de la Statistique et de la Démographie (ANSD) [Sénégal], et ICF International. Enquête Démographique et de Santé Continue (EDS-Continue 2012-2013). Calverton, Maryland, USA: ANSD et ICF International; 2013.
Serbia	Yes	Yes	2000	8	2012	26.8	13	European Health for All Database (HFA-DB) [online database updated on April 2014]. WHO Regional Office for Europe; 2014 (http://data.euro.who.int/hfadb/ , accessed 15 April 2015).

Country	Cross sectional analysis (n=150)	Trend analysis (n=121)	First year	First CS (%)	Last year	Last CS (%)	Data points (N)	SOURCES
Sierra Leone	Yes	No	-	-	2013	2.9	1	• Statistics Sierra Leone (SSL) and ICF Macro. Sierra Leone Demographic and Health Survey 2008. Calverton, Maryland, USA: Statistics Sierra Leone (SSL) and ICF Macro; 2009.<br>• Statistics Sierra Leone and UNICEF-Sierra Leone. Sierra Leone Multiple Indicator Cluster Survey 2010, Final Report. Freetown, Sierra Leone: Statistics Sierra Leone and UNICEF-Sierra Leone; 2011.<br>• Statistics Sierra Leone (SSL) and ICF International. Sierra Leone Demographic and Health Survey 2013. Freetown, Sierra Leone and Rockville, Maryland, USA: SSL and ICF International; 2014.
Slovakia	Yes	Yes	1990	8.7	2009	24.6	19	European Health for All Database (HFA-DB) [online database updated on April 2014]. WHO Regional Office for Europe; 2014 (http://data.euro.who.int/hfadb/, accessed 15 April 2015).
Slovenia	Yes	Yes	1990	8.57	2011	19.6	22	European Health for All Database (HFA-DB) [online database updated on April 2014]. WHO Regional Office for Europe; 2014 (http://data.euro.who.int/hfadb/, accessed 15 April 2015).
Solomon Islands	Yes	No	-	-	2007	6.2	1	National Statistics Office, Secretariat of the Pacific Community, Macro International Inc. Solomon Islands Demographic and Health Survey 2006-2007. Secretariat of the Pacific Community, Noumea, New Caledonia; 2009.
Spain	Yes	Yes	1990	14.22	2011	24.91	22	European Health for All Database (HFA-DB) [online database updated on April 2014]. WHO Regional Office for Europe; 2014 (http://data.euro.who.int/hfadb/, accessed 15 April 2015).
Sri Lanka	Yes	Yes	2003	20	2012	30.5	6	• Ministry of Healthcare and Nutrition. Department of Health. Medical Statistics Unit. Annual Health Statistics 2003. Sri Lanka, 2003.<br>• Ministry of Healthcare and Nutrition. Department of Health. Medical Statistics Unit. Annual Health Statistics 2005. Sri Lanka, 2005.<br>• Ministry of Healthcare and Nutrition. Department of Health. Medical Statistics Unit. Annual Health Statistics 2006. Sri Lanka, 2006.<br>• Department of Census and Statistics, Ministry of Health Care and Nutrition. Sri Lanka Demographic and Health Survey 2006-2007. Colombo, Sri Lanka, 2008.<br>• Ministry of Health. Annual Health Bulletin 2008, Sri Lanka, 2009.<br>• Medical Statistics Unit, Ministry of Health. Annual Health Bulletin 2012. Sri

Country	Cross sectional analysis (n=150)	Trend analysis (n=121)	First year	First CS (%)	Last year	Last CS (%)	Data points (N)	SOURCES
								Lanka; 2013. (only government institutions)
Sudan	Yes	Yes	1993	3.7	2010	6.6	3	• Pan Arab for Child Development (PAPCHILD). Sudan Maternal and Child Health Survey 1992/93. Federal Ministry of Health National Directorate of Motherhood, Childhood & Family Planning National Centre of Health Information. Republic of Sudan, League of Arab States, 1995.<br>• Ministry of Health, Government of Southern Sudan (MOH-GOSS), the Southern Sudan Commission for Census, Statistics and Evaluation (SSCCSE). Southern Sudan Household Health Survey (SHHS) 2006 (http://www.southsudanmedicaljournal.com/assets/files/misc/SHHS.pdf , accessed 20 April 2015)<br>• Federal Ministry of Health and Central Bureau of Statistics. Sudan Household and Health Survey - 2, 2012, National report. Khartoum, Republic of Sudan: Federal Ministry of Health and Central Bureau of Statistics; 2012.
Suriname	Yes	No	-	-	2010	19	1	Ministry of Social Affairs and Housing and General Bureau of Statistics. Suriname Multiple Indicator Cluster Survey 2010, Final Report: Paramaribo, Suriname; 2012.
Swaziland	Yes	No	-	-	2010	12.3	1	• Central Statistical Office (CSO) [Swaziland], and Macro International Inc. Swaziland Demographic and Health Survey 2006-07. Mbabane, Swaziland: Central Statistical Office and Macro International Inc.; 2008.<br>• Central Statistical Office, UNICEF. Swaziland Multiple Indicator Cluster Survey 2010. Final Report. Mbabane, Swaziland: Central Statistical Office, UNICEF; 2011.
Sweden	Yes	Yes	1990	10.4	2011	16.2	21	European Health for All Database (HFA-DB) [online database updated on April 2014]. WHO Regional Office for Europe; 2014 (http://data.euro.who.int/hfadb/ , accessed 15 April 2015).
Switzerland	Yes	Yes	1990	18.6	2009	32.2	13	European Health for All Database (HFA-DB) [online database updated on April 2014]. WHO Regional Office for Europe; 2014 (http://data.euro.who.int/hfadb/ , accessed 15 April 2015).
TFYR Macedonia	Yes	Yes	1995	1.4	2010	22.2	13	European Health for All Database (HFA-DB) [online database updated on April 2014]. WHO Regional Office for Europe; 2014 (http://data.euro.who.int/hfadb/

Country	Cross sectional analysis (n=150)	Trend analysis (n=121)	First year	First CS (%)	Last year	Last CS (%)	Data points (N)	SOURCES
								, accessed 15 April 2015).
Tajikistan	Yes	Yes	1990	1.9	2012	4.6	23	European Health for All Database (HFA-DB) [online database updated on April 2014]. WHO Regional Office for Europe; 2014 (http://data.euro.who.int/hfadb/ , accessed 15 April 2015).
Thailand	Yes	Yes	1990	15.2	2012	32	10	• Hanvoravongchai P, Letiendumrong J, Teerawattanon Y, Tangcharoensathien V. Implications of private practice on the caesarean section rate in Thailand. HRHDJ. 2000;4:2–12.<br>• Thailand Multiple Indicators Cluster Survey 2012. National Statistical Office, UNICEF, Ministry of Public Health, National Health Security Office, Thai Health Promotion Foundation, International Health Policy Program; 2013.
Timor-Leste	Yes	No	-	-	2009	1.7	1	National Statistics Directorate (NSD) [Timor-Leste], Ministry of Finance [Timor-Leste], and ICF Macro. Timor-Leste Demographic and Health Survey 2009-10. Dili, Timor-Leste: NSD [Timor-Leste] and ICF Macro; 2010.
Togo	Yes	Yes	1998	2	2010	8.8	2	• Anipah, Kodjo, Gora Mboup, Afi Mawuèna Ouro-Gnao, Bassanté Boukpessi, Pierre Adadé Messan et Rissy Salami-Odjo. Enquête Démographic et de Santé, Togo 1998. Calverton, Maryland USA: Direction de la Statistique et Macro International Inc.; 1999.<br>• Direction Générale de la Statistique et de la Comptabilité Nationale (DGSCN). Enquête par grappes à indicateurs multiples MICS Togo, 2010, Rapport final. Togo; 2012.
Trinidad and Tobago	Yes	No	-	-	2007	18.4	1	Mungrue K, Nixon C, David Y, Dookwah D, Durga S, Greene K, Mohammed H. Trinidadian women's knowledge, perceptions, and preferences regarding cesarean section: How do they make choices? Int J Womens Health. 2010;2:387–391.
Tunisia	Yes	Yes	1995	8	2012	26.7	4	• Ministère de la Santé Publique. Office National de la Famille et de la Population. L'Enquête Tunisienne sur la Santé de la Mère et de l'Enfant, Rapport Principal. Tunesie; 1996.<br>• Ministère de la Santé Publique et UNICEF. Enquête sur la Santé et le Bien-être de la Mère et l'Enfant 2000 (MICS 2). Tunis, Tunesie; 2000.<br>• Ministère de la Santé Publique et UNICEF. Enquête sur la Santé et le Bien-être de la Mère et l'Enfant 2006 (MICS 3). Tunesie; 2008.

Country	Cross sectional analysis (n=150)	Trend analysis (n=121)	First year	First CS (%)	Last year	Last CS (%)	Data points (N)	SOURCES
								• Ministère du Développement et de la Coopération Internationale, MDCI - Institut National de la Statistique et Fonds des Nations Unies pour l'Enfance. Suivi de la situation des enfants et des femmes en Tunisie- Enquête par grappes à indicateurs multiples 2011-2012, Rapport Final; 2013.
Turkey	Yes	Yes	1993	8	2011	47.5	7	European Health for All Database (HFA-DB) [online database updated on April 2014]. WHO Regional Office for Europe; 2014 (http://data.euro.who.int/hfadb/, accessed 15 April 2015).
Turkmenistan	Yes	Yes	1991	3.0	2012	6.6	20	European Health for All Database (HFA-DB) [online database updated on April 2014]. WHO Regional Office for Europe; 2014 (http://data.euro.who.int/hfadb/, accessed 15 April 2015).
Uganda	Yes	Yes	1995	2.6	2011	5.3	4	• Statistics Department [Uganda] and Macro International Inc. Uganda Demographic and Health Survey, 1995. Calverton, Maryland: Statistics Department [Uganda] and Macro International Inc.; 1996.<br>• Uganda Bureau of Statistics (UBOS) and ORC Macro. Uganda Demographic and Health Survey 2000-2001. Calverton, Maryland, USA: UBOS and ORC Macro; 2001.<br>• Uganda Bureau of Statistics (UBOS) and Macro International Inc. Uganda Demographic and Health Survey 2006. Calverton, Maryland, USA: UBOS and Macro International Inc.; 2007.<br>• Uganda Bureau of Statistics (UBOS) and ICF International Inc. Uganda Demographic and Health Survey 2011. Kampala, Uganda: UBOS and Calverton, Maryland: ICF International Inc.; 2012.
Ukraine	Yes	Yes	1990	5.7	2011	15.8	22	European Health for All Database (HFA-DB) [online database updated on April 2014]. WHO Regional Office for Europe; 2014 (http://data.euro.who.int/hfadb/, accessed 15 April 2015).
United Arab Emirates	Yes	Yes	1995	15.8	2013	23.9	4	• Fikri M, Farid SM. United Arab Emirates Family Health Survey 1995. Ministry of Health, Abu Dhabi, 2000.<br>• Ministry of Health. Statistics 2006. Abu Dhabi, United Arab Emirates; 2007.<br>• Ministry of Health. Statistics 2007. Abu Dhabi, United Arab Emirates; 2008.<br>• Ministry of Health. Statistics 2013. Abu Dhabi, United Arab Emirates; 2014.
United Kingdom	Yes	Yes	1990	11.3	2011	24.1	17	European Health for All Database (HFA-DB) [online database updated on April 2014]. WHO Regional Office for Europe; 2014 (http://data.euro.who.int/hfadb/

Country	Cross sectional analysis (n=150)	Trend analysis (n=121)	First year	First CS (%)	Last year	Last CS (%)	Data points (N)	SOURCES
								, accessed 15 April 2015).
United Republic of Tanzania	Yes	Yes	1996	2.1	2010	4.5	4	• Bureau of Statistics [Tanzania] and Macro International Inc. Tanzania Demographic and Health Survey 1996. Calverton, Maryland: Bureau of Statistics and Macro International; 1997.<br>• National Bureau of Statistics [Tanzania] and Macro International Inc. Tanzania Reproductive and Child Health Survey 1999. Calverton, Maryland, USA: National Bureau of Statistics and Macro International Inc.; 2000.<br>• National Bureau of Statistics (NBS) [Tanzania] and ORC Macro. Tanzania Demographic and Health Survey 2004-05. Dar es Salaam, Tanzania: National Bureau of Statistics and ORC Macro; 2005.<br>• National Bureau of Statistics (NBS) [Tanzania] and ICF Macro.Tanzania Demographic and Health Survey 2010. Dar es Salaam, Tanzania: NBS and ICF Macro; 2011.
United States of America	Yes	Yes	1990	22.7	2012	32.8	22	Martin JA, Hamilton BE, Osterman JK, Curtin SC, Mathews TJ. Births: Final Data for 2012. National Vital Statistics Reports. 2013;62(9).
Uruguay	Yes	Yes	1996	22	2012	39.9	16	Ministerio de Salud Pública,Dirección General de la Salud,División Epidemiología,Estadísticas Vitales - tipo de parto 1996-2012. Uruguay; 2014 (http://www.msp.gub.uy/) accessed on 20 April 2015
Uzbekistan	Yes	Yes	1995	1.9	2012	10.5	18	European Health for All Database (HFA-DB) [online database updated on April 2014]. WHO Regional Office for Europe; 2014 (http://data.euro.who.int/hfadb/ , accessed 15 April 2015).
Venezuela	Yes	No	-	-	2009	32.2	1	Ministerio del Poder Popular para la Salud (MPPS). Anuario Estadistico 2009. Venezuela;2010. (Only Ministry of Health accredited hospitals).
Viet Nam	Yes	Yes	1997	3.4	2014	27.5	4	• National Committee for Population and Family Planning. The Population and Family Health Project. Viet Nam Demographic and Health Survey 1997. Ha Noi, Viet Nam; 1999.<br>• Committee for Population, Family and Children [Vietnam], and ORC Macro. Vietnam Demographic and Health Survey 2002. Calverton, Maryland, USA: Committee for Population, Family and Children and ORC Macro; 2003.

Country	Cross sectional analysis (n=150)	Trend analysis (n=121)	First year	First CS (%)	Last year	Last CS (%)	Data points (N)	SOURCES
								• General Statistical Office (GSO), Viet Nam Multiple Indicator Cluster Survey - Final Report, 2011, Ha Noi, Viet Nam; 2011.<br>• General Statistical Office and UNICEF. Viet Nam Multiple Indicator Cluster Survey 2014, Key Findings. Ha Noi, Viet Nam; 2014.
Yemen	Yes	Yes	1997	1.4	2013	4.8	3	• Central Statistical Organization (CSO) [Yemen]] and Macro International Inc. (MI). Yemen Demographic and Maternal and Child Health Survey 1997. Calverton, Maryland, USA: CSO and MI; 1998.<br>• The Republic of Yemen Ministry of Health and Population, Central Statistical Organization and League of Arab States. The Yemen Family Health Survey: Principal Report. Pan Arab Project for Family Health. Cairo, Egypt: The Republic of Yemen Ministry of Health and Population, Central Statistical Organization and League of Arab States; 2004.<br>• Ministry of Public Health and Population, Central Statistical Organization, Measure DHS, ICF International. Yemen National Health and Demographic Survey 2013. Preliminary Report. ICF International, Rockville, Maryland, USA; 2014.
Zambia	Yes	Yes	1992	2.6	2007	3	4	• Central Statistical Office [Zambia], University of Zambia and Macro International Inc. Zambia Demographic and Health Survey, 1992. Macro International Inc., Columbia, Maryland, USA; 1993.<br>• Central Statistical Office [Zambia] and Ministry of Health and Macro International Inc. Zambia Demographic and Health Survey, 1996. Calverton, Maryland: Central Statistical Office and Macro International Inc.; 1997.<br>• Central Statistical Office [Zambia], Central Board of Health [Zambia], and ORC Macro. Zambia Demographic and Health Survey 2001-2002. Calverton, Maryland, USA: Central Statistical Office, Central Board of Health, and ORC Macro; 2003.<br>• Central Statistical Office (CSO), Ministry of Health (MOH), Tropical Diseases Research Centre (TDRC), University of Zambia, and Macro International Inc. Zambia Demographic and Health Survey 2007. Calverton, Maryland, USA: CSO and Macro International Inc.; 2009.

Country	Cross sectional analysis (n=150)	Trend analysis (n=121)	First year	First CS (%)	Last year	Last CS (%)	Data points (N)	SOURCES
Zimbabwe	Yes	Yes	1994	6	2014	6	5	• Central Statistical Office [Zimbabwe] and Macro International Inc. Zimbabwe Demographic and Health Survey, 1994. Calverton, Maryland, USA: Central Statistical Office and Macro International Inc.; 1995.<br>• Central Statistical Office [Zimbabwe] and Macro International Inc. Zimbabwe Demographic and Health Survey 1999. Calverton, Maryland, USA: Central Statistical Office and Macro International Inc.; 2000.<br>• Central Statistical Office (CSO) [Zimbabwe] and Macro International Inc. Zimbabwe Demographic and Health Survey 2005-06. Calverton, Maryland, USA: CSO and Macro International Inc.; 2007.<br>• Zimbabwe National Statistics Agency (ZIMSTAT) and ICF International. Zimbabwe Demographic and Health Survey 2010-11. Calverton, Maryland: ZIMSTAT and ICF International Inc.; 2012.<br>• Zimbabwe National Statistics Agency (ZIMSTAT). Multiple Indicator Cluster Survey 2014, Key Findings. Harare, Zimbabwe: ZIMSTAT: 2014.

* Not included in the cross-sectional analysis because the latest data available predated 2005.

# Not included in the trend analysis because inclusion criteria was: countries with a minimum of two data points over a time span of 10 years.
