# Supplementary material for: The Increasing Trend in Caesarean Section Rates: Global, Regional and National Estimates: 1990-2014
Source: PLoS One. 2016 Feb 5;11(2):e0148343. doi: 10.1371/journal.pone.0148343 (PMC4743929; doi:10.1371/journal.pone.0148343)
Supplement: S2 File — Data set compiled for the analysis of caesarean section rates and trends. (PDF) [file pone.0148343.s002.pdf]

Country	UN_region	UN_subreg	cs1980	cs1981	cs1982	cs1983	cs1984	cs1985	cs1986	cs1987	cs1988	cs1989	cs1990	cs1991	cs1992	cs1993	cs1994	cs1995	cs1996	cs1997	cs1998	cs1999	cs2000	cs2001	cs2002	cs2003	cs2004	cs2005	cs2006	cs2007	cs2008	cs2009	cs2010	cs2011	cs2012	cs2013	cs2014
World																																					
More developed regions																																					
Less developed regions																																					
Least developed countries																																					
Africa																																					
Eastern Africa																																					
Burundi	AFRI	EAF																															4				
Comoros	AFRI	EAF																	5.3																9.6		
Djibouti	AFRI	EAF																																			
Eritrea	AFRI	EAF																1.6							2.7								2.8				
Ethiopia	AFRI	EAF																					0.6					1						1.5			
Kenya	AFRI	EAF														5.2					6.8					4					6.2						
Madagasca	AFRI	EAF													1					0.6						1					1.5					1.9	
Malawi	AFRI	EAF													3.4								2.8				3.1						4.6				5.1
Mauritius	AFRI	EAF																																			
Mozambiqu	AFRI	EAF																		2.7						1.9								3.9			
Réunion	AFRI	EAF																																			
Rwanda	AFRI	EAF													1.8								2.1					2.9					7.1				
Somalia	AFRI	EAF																																			
Uganda	AFRI	EAF																2.6					2.5						3.1					5.3			
United Re	AFRI	EAF																	2.1			2.9					3.2						4.5				
Zambia	AFRI	EAF													2.6				1.9					2.1						3							
Zimbabwe	AFRI	EAF															6					6.7						4.8					4.5				6
Middle Africa																																					
Angola	AFRI	MAF																																			
Cameroon	AFRI	MAF												2.4							2.5						2							3.8			
Central A	AFRI	MAF															1.9																4.5				
Chad	AFRI	MAF																	0.5								0.4						1.5				
Congo	AFRI	MAF																																5.8			
Dem. Rep.	AFRI	MAF																												4			7.2				
Equatoria	AFRI	MAF																																6.6			
Gabon	AFRI	MAF																					5.6												10		
Northern Africa																																					
Algeria	AFRI	NAF													6.3																				16.3		
Egypt	AFRI	NAF													4.6			6.6					10.3					19.9			27.6						51.8
Libyan Ar	AFRI	NAF																7.2																			
Morocco	AFRI	NAF													2			3.5								5.4								16			
Sudan	AFRI	NAF														3.7													4.5				6.6				
Tunisia	AFRI	NAF																8					17.8						20.5						26.7		
Western S	AFRI	NAF																																			
Southern Africa																																					
Botswana	AFRI	SAF																						7.7													
Lesotho	AFRI	SAF																									5.1					6.7					
Namibia	AFRI	SAF													6.9														12.7							14.4	
South Afr	AFRI	SAF																			15.5					20.6											
Swaziland	AFRI	SAF																											7.9				12.3				
Western Africa																																					
Benin	AFRI	WAF																	2.2					3.3					3.6					5.4			
Burkina F	AFRI	WAF														1.3					1.1					0.7							1.9				
Cape Verd	AFRI	WAF																										10.7									
C?te d'Iv	AFRI	WAF															1.8				2.5							6.4						2.7			
Gambia	AFRI	WAF																															2.5				
Ghana	AFRI	WAF														4.4					4					3.7					6.9			11.4			
Guinea	AFRI	WAF													3.3							2						1.7							2.4		
Guinea-Bi	AFRI	WAF																															2.3				
Liberia	AFRI	WAF																												3.5							
Mali	AFRI	WAF																0.8						1.1					1.6							2.7	
Mauritani	AFRI	WAF																						3.3										9.6			
Niger	AFRI	WAF													0.9						0.6								1						1.4		
Nigeria	AFRI	WAF											2.9									3.7				1.7					1.8					2	
Senegal	AFRI	WAF													2.3													3.3					5.9		3.8		
Sierra Le	AFRI	WAF																													1.5		4.5			2.9	
Togo	AFRI	WAF																			2												8.8				
Asia																																					
Eastern Asia																																					
China	ASIA	EAS									3.697	4.278	4.363	5.878	6.986	7.105	7.337	7.996	9.035	10.922	14.069	15.673	22.029	25.401	25.904	27.848	32.693	35.109	37.348	39.287	44.815			36.2			
China, Ho	ASIA	EAS																				27.4															
China, Ma	ASIA	EAS																																			
Dem. Peop	ASIA	EAS																														12.5					
Japan	ASIA	EAS								8.46			10.03			11.83			12.56			14.74			15.19			17.36			18.41			19.22			
Mongolia	ASIA	EAS																			5.1					9.6					17.4		21			23.4	
Republic	ASIA	EAS												17.3			31.5			35.9			33.7			39.2			35			36.6					
South-central Asia																																					
Afghanist	ASIA	SCAS																																3.6			
Banglades	ASIA	SCAS																				2.4					3.5			7.5				17.9		19.1	
Bhutan	ASIA	SCAS																															12.4				
India	ASIA	SCAS													2.5						7.1							8.5			8.2						
Iran (Isl	ASIA	SCAS																					35					38.4		45		47.9					
Kazakhsta	ASIA	SCAS				1.446	1.902	2.309	2.768	3.299	3.677	4.084	4.641	5.022	5.289	5.101	5.086	5.194	5.54	6.034	6.497	6.794	6.837	7.417	8.143	9.272	93.8	10.059	10.664	11.684	12.448	13.611	13.532	14.62	15.2		
Kyrgyzsta	ASIA	SCAS	0.825	0.915	0.975	0.957	1.195	1.428	1.659	1.928	2.331	2.634	3.054	2.99	3.942	3.114	3.214	3.078	3.544	3.887	3.804	3.994	3.859	4.085	4.021	4.237	5.09	5.061	5.099	5.836	6.443	6.662	6.737		5.8		7.4
Maldives	ASIA	SCAS																														32.4		41.1			
Nepal	ASIA	SCAS																	1					0.8					2.7					4.6			
Pakistan	ASIA	SCAS												2.7															7.3						14.1		
Sri Lanka	ASIA	SCAS																								20		22.1	23.6	24.5	25.7				30.5		
Tajikista	ASIA	SCAS											1.863	2.06	2.099	1.567	1.29	1.238	1.671	1.297	1.335	1.207	1.494	1.394	1.649	1.562	1.863	2.043	2.1	2.45	2.772	3.353	3.57	4.07	4.57		
Turkmenis	ASIA	SCAS												3.01	2.699	2.845	2.53	2.49	2.626	2.883			3.206	3.093	3.354	3.003	2.543	3.199	3.353	3.802	3.916	4.485	4.68	5.86	6.64		
Uzbekista	ASIA	SCAS																1.937	2.167	2.278	2.433	2.716	3.104	3.317	3.588	3.773	4.377	4.938	6.115	6.287	6.687	7.748	8.642	9.04	10.54		
South-eastern Asia																																					
Brunei Da	ASIA	SEAS																																			
Cambodia	ASIA	SEAS																					0.8					1.8					3				
Timor-Les	ASIA	SEAS																														1.7					
Indonesia	ASIA	SEAS												1.3			2.5			4.3					4.1					6.8					12.3		
Lao Peopl	ASIA	SEAS																															2		3.7		
Malaysia	ASIA	SEAS																											15.7								

Myanmar	ASIA	SEAS																																			
Philippin	ASIA	SEAS														5.9					5.7					7.3					9.5			11.1		9.3	
Singapore	ASIA	SEAS																																			
Thailand	ASIA	SEAS											15.2	16	17	18.4	20	21.1	22.4					17.4								24.3			32		
Viet Nam	ASIA	SEAS																		3.4					9.9									20			27.5
Western Asia																																					
Armenia	ASIA	WAS	0.91	1.015	1.109	1.224	1.494	1.677	2.021	2.167	2.609	3.006	3.364	3.586	3.661	3.77	3.697	4.275	4.812	5.652	6.178	6.586	7.448	7.859	8.468	9.125	9.624	11.328	11.25	14.058	15.137	17.747	18.827	21.7	22.5		
Azerbaija	ASIA	WAS											1.44	1.595	1.702	1.682	1.826	1.883	2.005	1.999	2.067	2.504	2.579	3.104	3.568	4.136	4.703	5.279	6.146	7.616	10.288	12.666	13.732	14.74	16.74		
Bahrain	ASIA	WAS																16									16.2	19.8	20.9	23.4	23.9	23.6	26.8	30	26		
Cyprus	ASIA	WAS																										6.866	6.746	7.603	8.278	9.502	11.43				
Georgia	ASIA	WAS											3.81	4.633	5.136	4.588	5.524	5.841	6.815	7.071	8.191	7.526	9.12	10.357	11.81	13.549	15.721	19.507	20.785	22.334	24.52	27.963	31.065	34.72	36.7		
Iraq	ASIA	WAS																											20.6					22.2			
Israel	ASIA	WAS								8.032		9.295	9.872			10.915	11.621	12.173	12.677	12.79	13.397	14.594	15.776	16.662	17.252	17.5	17.607	18.613	18.744	18.871	19.492	19.22	19.151	19.78			
Jordan	ASIA	WAS											5.7							10.5					16					18.5					28		
Kuwait	ASIA	WAS																	11.2																		
Lebanon	ASIA	WAS																15.1				23.3					23.2										
Occupied	ASIA	WAS																	6.1																		
Oman	ASIA	WAS																6.6					8.2			11	11.52	12.62	13.48	14.1	14.89		16.38	16.98			
Qatar	ASIA	WAS																			15.9														19.5		
Saudi Ara	ASIA	WAS																	8.1					11.6					18.5	19.2	20.4	21.2	22.3		22.3		
Syrian Ar	ASIA	WAS																					14.8														
Turkey	ASIA	WAS														8					13.6									37.498	40.89	44.488	47.221	47.52			
United Ar	ASIA	WAS																15.8											18.5	20.7						23.9	
Yemen	ASIA	WAS																		1.4												6.5				4.8	
Europe																																					
Eastern Europe																																					
Belarus	EURO	EE														9.207	9.756	10.992	12.067	13.886	15.202	15.595	16.69	16.397	17.424	17.773	18.281	18.911	19.543	20.487	21.042	21.957	22.986	24.05	25.32		
Bulgaria	EURO	EE	4.709	5.41	5.59	6.454	6.406	6.801	7.359	7.777	7.658	7.47	7.274	7.139	7.821	8.498	9.209	10.034	10.304	10.983	12.439	13.144	13.473	14.701	16.821	17.505	19.174	22.023	23.626	26.791	28.442	32.808	31.034	33.12			
Czech Rep	EURO	EE	4.408	4.783	5.289	5.716	5.965	6.268	6.46	6.976	7.52	7.55	7.611	8.351		9.099	10.09	11.202	11.558	11.789	12.265	12.339	12.892	13.247	14.067	15.29	16.008	17.133	18.368	15.565	20.478	21.22	22.488	23.27			
Hungary	EURO	EE															12.556	13.607	14.551	15.677	17.012	18.722	20.149	21.18	23.196	24.746	25.741	27.919	28.116	28.576	29.872	31.327	32.506	33.41			
Poland	EURO	EE															13.777	15.175	15.559	16.122											29.961	31.628	33.108	29.89			
Republic	EURO	EE	1.982	2.148	2.403	2.537	2.869	3.419	3.541	4.125	4.34	4.718	5.262	5.288	4.982	5.366	5.323	5.602	5.979	6.836	7.33	6.525	7.548	6.736	7.708	8.497	8.847	9.837	11.052	11.895	13.309	13.161	13.89	14.58	15.83		
Romania	EURO	EE													7.201	8.625	9.08	10.093	10.457	11.057	11.977	12.476	14.078	15.625	16.654	17.73	18.937	21.38	21.537	23.644	25.841	28.635	33.82	36.31			
Russian F	EURO	EE											6.72	7.19	8.066	9.009	9.361	9.862	10.839	11.849	12.575	13.133	13.955	14.76	15.163	15.663	16.157	17.206	17.99				22.07				
Slovakia	EURO	EE						6.269	6.715	8.274		8.343	8.707	9.117	9.917	10.585		11.604	12.238	12.967	13.285	13.845	14.642	16.654	17.645	18.125	18.794	20.013	20.902	22.27	23.272	24.609					
Ukraine	EURO	EE	1.471	1.715	1.962	2.207	2.462	2.898	3.344	4.072	4.648	5.177	5.711	6.156	5.112	6.98	7.166	7.503	8.074	8.573	9.149	9.513	10.44	11.414	12.19	12.759	13.359	13.6387	14.201	15.223	15.555	15.93	15.84	15.82			
Northern Europe																																					
Channel I	EURO	NE																																			
Denmark	EURO	NE	10.4	11.388	12.2	12.135	12.612	13.001	13.196	12.867	13.04	12.616	12.577	12.297	12.226	12.737	12.63	12.55	12.882	13.65	14.514	15.026	15.05	16.629	18.285	19.208	20.184	19.729	20.45	21.161	20.749	21.354	20.798				
Estonia	EURO	NE											6.272	6.295	6.387	6.956	8.684	9.579	10.134	11.267	13.134	13.956	14.533	15.508	14.976	16.362	17.146	18.857	18.962	19.975	19.971	20.694	20.335	20.2			
Finland	EURO	NE			12.7		13.1	14.8	15.2	14.283	13.885	14.127	13.516	14.307	14.487	14.576	15.296	15.588	15.655	15.54	15.318	15.783	15.782	16.404	16.359	16.165	16.391	16.275	16.14	16.259	16.489	15.749	16.141	14.74			
Iceland	EURO	NE	7.4	10.3	10.1	11.4	11.2	11.229	11.827	12.998	11.962	11.469	11.808	11.427	13.43	12.979	13.755	14.112	15.154	16.261	15.917	17.317	17.683	16.769	17.362	17.91	16.415	15.561	17.214	16.908	16.215	16.176					
Ireland	EURO	NE		6.2			7.4	7.713	8.488	8.913	9.648	10.075	10.538	11.558	12.036	12.867		13.418	14.528	15.337	17.501	18.424	21.426	22.132	22.506	24.299	25.418	26.19	25.885	26.594	25.594	26.363	26.55				
Latvia	EURO	NE	3.996	4.212	4.595	4.562	4.911	4.807	5.038	5.943	5.41	6.212	7.044	7.764	7.5014	8.961	9.371	10.438	11.526	12.252	15.812	14.199	15.014	16.345	17.408	19.115	19.622	20.258	21.081	23.344	22.57	23.2714	23.638	22.99			
Lithuania	EURO	NE													8.251	9.283	9.975	10.64	10.766	11.221	11.5	11.987	12.829	13.514	15.146	14.913	16.759	18.29	19.226	20.488	21.129	21.215	21.423	23.21			
Norway	EURO	NE	8.382	8.784	9.037	9.426	10.898	11.969	12.088	12.919	12.784	12.942	12.747	12.482	12.559	12.475	12.599	12.642	12.727	12.878	13.665	13.449	13.661	15.654	16.057	16.058	15.7	16.876	16.627	16.991	17.465	17.289					
Sweden	EURO	NE	11.476	11.8	11.768	11.816	11.293	11.631	11.127	10.825	10.741	10.515	10.351	10.735	10.66	10.995	11.151	11.361	11.819	12.464	13.09	13.645	14.485	15.623	15.747	15.977	16.567	16.888	17.2747	17.076	16.685		16.44	16.22			
United Ki	EURO	NE	8.204		9.193	9.151	9.227	9.393					11.286					14.155		16.648	18.004	19.401	19.969	21.035	21.655	21.883	22.01	22.142	22.543	23.006	23.069	23.185	23.665	24.12			
Southern Europe																																					
Albania	EURO	SE													9.332	9.771	7.768	8.446	8.646	10.021	10.105	10.665	12.464	13.07	16.458	15.088	18.014	20.29	24.302	25.64	22.736	28.099	29.696	30			
Bosnia an	EURO	SE																														17.496	15		13.9		
Croatia	EURO	SE				1.134	1.468	1.867	2.034	3.06	2.987	4.314	5.329	5.765	5.614	6.329	6.346	7.951	7.987	11.097	12.015	11.578	10.828	11.912	14.025	14.16	14.906	16.001	16.195	16.359	17.05	17.527	18.75		20.16		
Greece	EURO	SE	18	18	17	16	16	18	15	20	21	22	24	24																							
Italy	EURO	SE	11.2	12.7	13.2	14.5	15.7	15.8	15.7	17.5	18.792	19.628	20.789	22.604	23.24	24.129	24.757	26.067	23.886	27.042	29.732	32.39	33.302	34.795	36.191	37.265	37.649	38.239	38.944	38.644	38.543	38.347	38.82	38.11			
Malta	EURO	SE																18.166	17.536	18.987	21.39	22.075	22.793	23.532	23.4	25.75	26.962	30.197	34.253	31.987	30.079	28.757	31.16	33.51			
Montenegr	EURO	SE																		9.032	10.162	10.014	9.887	14.232	12.778	15.125							23.564				
Portugal	EURO	SE	7.055	7.122	7.458	10.414	10.557	10.129	12.281	13.218	14.779	15.915	18.605	20.009	21.786	22.521	23.868	24.171	24.625	27.405	27.529		27.729	29.53	30.22	31.878	32.433	33.998	34.724	34.908	35.565	36.156	36.2				
Serbia	EURO	SE																					7.981	8.552	8.008	9.25	10.82	11.741	16.464	18.051	19.343	21.073	23.854	25.76	26.78		
Slovenia	EURO	SE		5.232	5.788	5.67	5.596	6.444	4.196	7.877	7.085	7.711	8.57	8.901	9.148	9.599	9.351	9.215	10.044	10.157	10.299	10.124	10.985	12.474	13.679	14.353	14.279	15.469	16.372	16.771	16.992	17.876	19.071	19.6			
Spain	EURO	SE				8.676	9.569	10.446	11.365	12.149	12.899	13.733	14.22	15.028	16.198	17.329	17.777	18.803	19.285	19.799	20.54	21.012	21.512	22.412	23.382	23.703	24.236	25.012	25.925	25.29	24.675	24.946	24.9	24.91			
TFYR Mace	EURO	SE																1.403		7.752	8.607	9.832	9.117	10.218	10.511	11.443			16.927	19.781	18.004	17.636	22.172				
Western Europe																																					
Austria	EURO	WE		6.5	7	7.5												12.385	13.094	13.947	14.578	16.424	17.201	18.861	20.614	22.116	23.555	24.419	25.811	27.146	28.003	28.803	28.884	28.32			
Belgium	EURO	WE	7.4	7.664	8.1	8.215	9.34	9.416	9.714	9.586	9.586	10.231	10.445	11.613	11.965	12.944	12.941	13.446	13.963	14.539	14.435	16.016											19.72				
France	EURO	WE																		16.3	16.94	17.45	17.88	18.59	19.24	19.35			20.24	20.71	20.64	20.8	21.03	21			
Germany	EURO	WE											15.7	15.216	15.942	16.574	17.067	17.24	17.611	18.138	19.072	19.801	20.884	21.995	23.67	24.81	25.952	26.735	27.781	28.404	29.37	30.291					
Luxembour	EURO	WE																	15.94	18.081	17.75	18.81	19.87	21.95	23.14	23.53	24.2	24.2	24.3	25.8	26.8	26.1	25.78	27.36			
Netherlan	EURO	WE						6.5					7.5	7.8	8.2	8.7		9.647	10.06	10.391	11.058	11.342	11.87	13.637	13.516	13.529	13.645	13.569	13.775	13.922	14.302		15.59				
Switzerla	EURO	WE				18	20.4	19.8	19.2	19.2	17.1	16.9	18.6	17.7								16.822	19.024	23.408	25.41	27.5	27.814	29.044	30.712	31.494	32.502	32.153					
Latin America and Caribbean																																					
Caribbean																																					
Bahamas	LACB	CBB																																			
Barbados	LACB	CBB																																			
Cuba	LACB	CBB																							28.5												
Dominican	LACB	CBB												20					25.9			27.9			31.3					41.9					43.47	56.4	
Guadeloup	LACB	CBB																																			
Haiti	LACB	CBB															1.6						1.6					3							5.5		
Jamaica	LACB	CBB																							9.6						14.8			21.2			
Martiniqu	LACB	CBB																																			
Netherlan	LACB	CBB																																			
Puerto Ri	LACB	CBB																34.25																			
Saint Luc	LACB	CBB																																			
Trinidad	LACB	CBB																												18.4							
Central America																																					
Belize	LACB	CAM												8.4								9.1												28.9			
Costa Ric	LACB	CAM	15.8	15.43	13.89	14.11	14.74	15.16	16.27	18	18.3	19.28	19.76	19.66	20.35	20.89	20.56	20.66	20.94	20.97	20.84	21.68	21.28	22.18	21.96	22.02	21.79	21.6	22.12	21.44	20.48	20.44	20.91	20.52	21.6	21.88	
El Salvad	LACB	CAM												13.4							15.7				22			27.6	28.1	28.2	28.1	29.9	29.5	30.4	28.2	29.8	
Guatemala	LACB	CAM																8.2			10.8				11.4						16.3						
Honduras	LACB	CAM												6.4				6.3						7.9				13						18.6			
Mexico	LACB	CAM								12.4													29.9			33.2			36.9			43.1			46.2		
Nicaragua	LACB	CAM														13.5					15.4			14.7					19.6							29.7	
Panama	LACB	CAM																														20.2				27.7	
South America																																					
Argentina	LACB	SAM																											22.7								
Bolivia	LACB	SAM										7.9					11				14.7					14.6					18.6						

Brazil	LACB	SAM						37.8	40.23	40.74	40.22	38.42	37.22	38.02	38.3	38.77	40.08	41.84	43.29	45.11	46.56	48.45	50.1	52.34		55.61	
Chile	LACB	SAM		25.4	26.9	28.2	28.6	29.1	29.7	29.8	31.2	31.6	30.3	30.4	30.7	30.7	30.9	30.9	32.4	33.7	34.7	35.7	36.4	37	38.4		
Colombia	LACB	SAM		16					16.9			24.85	26.07	26.87	27.22	28.26	28.6	29.37	31.26	33.17	35.17	37.17	38.96	40.13	42.78	43.4	
Ecuador	LACB	SAM						17.1					19.9					25.8									
French Gu	LACB	SAM																									
Guyana	LACB	SAM																					13.3				
Paraguay	LACB	SAM		13								16.5						26.9				33.1					
Peru	LACB	SAM				10				8.7				12.7					15.9		18.4		21.4	20.4	22.9		26.5
Suriname	LACB	SAM																						19			
Uruguay	LACB	SAM								21.98	21.7		22.8	23.9	26.1	25.8	26.6	28.9	30.97	31.23	33	33.36	35.55	38.12	40.09	39.87	
Venezuela	LACB	SAM														25.1							32.2				
Northern America																											
Canada	NOAM					18.2	17.9	17.8	17.5	17.6	18.2	18.5	19	19.7	21.2	22.5	23.6	24.8	25.6	26.3	27.3	27.7			26.9	27.1	
United St	NOAM		22.8	22.7	22.6	22.3	21.8	21.2	20.8	20.7	20.8	21.2	22	22.9	24.4	26.1	27.5	29.1	30.3	31.1	31.8	32.3	32.9	32.8		32.8	
Oceania																											
Australia/New Zeal		ANZ																									
Australia	OCEA	ANZ			18	18.3	19	19.4	19.3	19.5	20.3	21.1	21.9	23.3	25.4	27	28.5	29.4	30.3	30.8	30.9	31.1	31.5	31.6	32.3	32.4	
New Zeala	OCEA	ANZ								20.8	22.3	24.7	24.5	26.6		29.6	29.2	29.3	31.6	33.1	31.7	31.3	31.2	32.3	32.5	33.4	
Melanesia																											
Fiji	OCEA	MEL												7.1													
New Caled	OCEA	MEL																									
Papua New	OCEA	MEL														4.7											
Solomon I	OCEA	MEL																			6.2						
Vanuatu	OCEA	MEL																									
Micronesia		MIC																									
Guam	OCEA	MIC																									
Polynesia																											
French Po	OCEA	POL																									
Samoa	OCEA	POL																					12.8				
